# Role of chemokine-mediated angiogenesis in resistance towards crizotinib and its reversal by anlotinib in *EML4-ALK* positive NSCLC

**DOI:** 10.1186/s12967-022-03451-2

**Published:** 2022-05-31

**Authors:** Shasha Wang, Ning Lou, Rongrong Luo, Xuezhi Hao, Yutao Liu, Lin Wang, Yuankai Shi, Xiaohong Han

**Affiliations:** 1 Department of Clinical Laboratory, National Cancer Center/National Clinical Research Center for Cancer/Cancer Hospital, Chinese Academy of Medical Sciences & Peking Union Medical College, Beijing, 100021 China; 2 Department of Medical Oncology, National Cancer Center/National Clinical Research Center for Cancer/Cancer Hospital, Chinese Academy of Medical Sciences & Peking Union Medical College, Beijing Key Laboratory of Clinical Study On Anticancer Molecular Targeted Drugs, No. 17, Panjiayuan Nanli, Chaoyang District, Beijing, 100021 China; 3 Clinical Pharmacology Research Center, Peking Union Medical College Hospital, State Key Laboratory of Complex Severe and Rare Diseases, NMPA Key Laboratory for Clinical Research & Evaluation of Drug, Beijing Key Laboratory of Clinical PK & PD Investigation for Innovative Drugs, Chinese Academy of Medical Sciences & Peking Union Medical College, No.1, Shuaifuyuan, Dongcheng District, Beijing, 100730 China

**Keywords:** ALK-TKIs, Drug resistance, Chemokines, Angiogenesis, Anlotinib, Biomarkers

## Abstract

**Background:**

The identification of early plasma biomarkers for clinical outcomes and drug resistance has key importance for risk stratification in *anaplastic lymphoma kinase* (*ALK*)-positive advanced non-small cell lung cancer (NSCLC) patients. Moreover, it remains unclear whether the anti-angiogenic drug anlotinib can reverse the resistance of ALK-tyrosine kinase inhibitor (ALK-TKI) crizotinib, and no research has explored the effect of anlotinib combined with crizotinib on *ALK*-positive patients.

**Methods:**

In this study, 76 baseline and longitudinal plasma samples from 61 *ALK*-positive NSCLC patients receiving crizotinib treatment were analyzed by Luminex liquid suspension chip for 40 chemokines. RNA sequence (RNA-seq) was used to identify differentially expressed genes (DEGs) between H3122 and H3122-crizotinib resistant (H3122CR) cells. Tube formation assay was performed to investigate the effect of chemokines on angiogenesis. And H3122CR-derived xenograft model was constructed to validate the efficacy and safety of anlotinib combined with crizotinib in vivo.

**Results:**

Baseline and progression plasma samples detection suggested that CCL20 played a crucial role in monitoring and predicting the clinical response of crizotinib (hazard ratio for progression-free survival: 2.27 (1.13–4.58); for overall survival: 2.7 (1.23–5.8)). RNA-seq results for H3122 and H3122CR cells showed that high expression of chemokines and angiogenesis pathways were involved in crizotinib resistance. Subsequently, in vitro experiments indicated that CCL20 may induce crizotinib resistance by activation of angiogenesis via JAK2/STAT3-CCL20-VEGFA/IL6 axis. We further found that anti-angiogenic TKI anlotinib could reverse crizotinib resistance by inhibiting chemokines-induced angiogenesis, and anlotinib combined with crizotinib has a better antitumor effect than monotherapy in vitro & in vivo.

**Conclusions:**

Overall, CCL20-mediated angiogenesis is involved in crizotinib resistance and could be overcome by using anlotinib in *EML4-ALK* positive NSCLC. The combination of anlotinib and crizotinib is a promising strategy for patients resistant to ALK-TKIs.

**Supplementary Information:**

The online version contains supplementary material available at 10.1186/s12967-022-03451-2.

## Background

After the discovery of *echinoderm microtubule associated protein like 4 (EML4)-ALK* rearrangement in NSCLC, *ALK*-positive NSCLC patients obtained remarkably improved progression-free survival (PFS) and overall survival (OS) with ALK-TKIs treatment [1, 2]. However, due to the emergence of primary and acquired resistance to targeted therapy, clinical outcomes are heterogeneous among different patients [2, 3]. Illuminating the molecular mechanism of drug resistance and searching for prognostic biomarkers are conducive to guiding next-line therapies.

A compelling body of evidence indicates that angiogenesis is involved in the occurrence and development of many solid tumors, including lung cancer [4, 5]. Chemokines are small proteins (8–10 kDa) belonging to the family of chemoattractant cytokines, including 4 subtypes, C, CC, CXC, and CX3C [6]. The chemokine network has been reported to participate in regulating the distribution of immune cells and angiogenic activity in the tumor microenvironment [6, 7]. Among them, several studies demonstrated that the interaction of CCL20 and CCR6 promoted the tumor progression in melanoma, breast cancer, and hepatocellular carcinoma via enhancing angiogenesis [8, 9]. Moreover, CCL20 induced cell proliferation and migration in lung cancer[10]. The expression level of CCL20 in serum served as a crucial biomarker for prognostic prediction in NSCLC patients [11]. However, whether CCL20 can serve as a prognostic marker for *ALK*-positive NSCLC patients, and the role of CCL20-induced angiogenesis in crizotinib resistance remain unclear.

Anlotinib hydrochloride (AL3818) is a novel multi-target TKI to inhibit the angiogenesis of tumor [12, 13]. It has been recommended as a third-line or further treatment for driver gene-positive advanced NSCLC in China [14]. We hypothesized that anlotinib could improve the clinical outcomes via blocking the angiogenesis in *ALK*-positive NSCLC patients, but the underlying molecular mechanism has not been fully clarified yet. Furthermore, recent clinical trials indicated that the combination of anti-angiogenic drugs (such as bevacizumab or anlotinib) with chemotherapy, epidermal growth factor receptor (EGFR)-TKIs targeted therapy, or immunotherapy prolonged the PFS and OS of NSCLC patients [14–17]. However, whether the combination of anlotinib with ALK-TKIs can reverse crizotinib resistance and improve the response to ALK-TKIs has not been reported yet. In the current study, we identified plasma biomarkers to monitor and predict the drug resistance and clinical response of crizotinib, clarified its underlying mechanism, and explored the anti-tumor effect of the combination of anlotinib and crizotinib in crizotinib-resistant NSCLC.

## Methods

### Patient samples

76 plasma samples were collected from 61 *EML4-ALK* positive NSCLC patients treated with crizotinib in the Cancer Hospital, Chinese Academy of Medical Sciences & Peking Union Medical College (CAMS & PUMC) from 2010 to 2015 (CHCAMS cohort). All baseline samples of 61 patients were obtained before the initiation of treatment, and the progression samples of 15 patients with PFS longer than 12 months were collected after crizotinib resistance. The cut-off date for the follow-up was February 2, 2021. Peripheral blood samples were collected in K_2_EDTA tubes and centrifuged at 1600 g for 10 min after collecting. The isolated plasma was centrifuged again at 16,000 g for 10 min to remove cell debris. All extracted plasma samples were stored at – 80 ℃. All samples and data collected were with informed consent. This study was approved by the medical ethics committee of Cancer Hospital, CAMS & PUMC (No.19-019/1804). The patient characteristics of the CHCAMS cohort were shown in Table 1.

**Table 1:** Patient characteristics

All *ALK*^+^ NSCLC patients analyzed in this study (n = 61)		
Age, median (SD)		49 (13)
Sex, % female		65.6%
Smoking status (% never smokers)^a^		73.2%
ECOG PS (%) at baseline^b^	0	8
	1	31
	2	1
Histology (%)	Adenocarcinoma	49/50^c^
Chemotherapy		52/61
ALK TKI, patient number	Crizotinib	61
Metastasis	Brain^d^	33/49
	Bone^e^	31/55
Follow-up in months (median, [IQR])		52.4 (38.0–82.43)
Cases with baseline samples		61
Cases with disease progression samples		15

*ALK* anaplastic lymphoma kinase, *NSCLC* non-small cell lung cancer, *SD* standard deviation, *PS* performance status, *TKI* tyrosine kinase inhibitor, *IQR* interquartile range

^a^Data available for 41/61 cases

^b^Data available for 40/61 cases

^c^Data available for 50/61 cases, one patient has a lung squamous carcinoma

^d^Data available for 49/61 cases

^e^Data available for 55/61 cases

### Cells lines and reagents

H3122 (*EML4-ALK*, variant 1) and H2228 (*EML4-ALK*, variant 3) cell lines were purchased from ATCC and PUMC-HUVEC-T1 cells were obtained from Cell Resource Center, Peking Union Medical College (Beijing, China). Crizotinib-resistant cell line (H3122CR) was developed from its parental cell line H3122 in our previous study [18]. RPMI-1640 medium (HyClone, USA) with 10% FBS (Gibco, USA) was used to culture H3122 and H2228 cells. PUMC-HUVEC-T1 cells were cultured in DMEM containing 10% FBS and 1% NEAA (Cell Resource Center, Beijing). Additionally, crizotinib and STAT3 inhibitor Stattic were purchased from Sigma-Aldrich and MedChemExpress (MCE), respectively. Anlotinib was obtained from Chiatai Tianqing (Jiangsu, China).

### Luminex liquid suspension chip detection

The expression levels of 40 chemokines in plasma samples and cell supernatants were detected using the Bio-Plex Pro Human Chemokine Panel 40-plex kit with Luminex 200 system performed by Wayen Biotechnologies (Shanghai, China). The 40 chemokines evaluated are shown in Additional file 5: Table S1.

### RNA-seq and DEGs analysis

Total RNA from H2228, H3122, and H3122CR cells cultured for 48 h was extracted using the TRIzol reagent. The NEBNext Ultra RNA Library Prep Kit (NEB, USA) was used to construct cDNA library following the manufacturer’s directions. Paired-end sequencing with 150 bp reads was conducted on the Illumina Novaseq platform (Novogene, Beijing, China). After data cleaning, all clean reads were mapped to the reference genome (hg38) using Hisat2 v2.0.5. The gene expression level was measured by the fragments per kilobase per million (FPKM). The edgeR R package (3.18.1) was used to identify DEGs between H3122 and H3122CR cells. We selected DEGs with a corrected p-value < 0.05 and log2^Fold Change^ > 1. The clusterProfiler R package was used to perform the Gene Ontology (GO) enrichment analysis and Gene Set Enrichment Analysis (GSEA) for DEGs.

### RT-qPCR

Total RNA isolation was performed in cell lines after treatment for 24 h using the RNeasy Mini Kit (Qiagen, Germany). cDNA was synthesized by reverse transcription polymerase chain reaction (RT-PCR) with PrimeScript ™ RT reagent kit (TAKARA, Japan). Then the SYBR Premix Ex Taq ™ II kit (TAKARA, Japan) was used for quantitative real-time PCR (qPCR) assay on Roche LightCycler480 II platform. GAPDH was used as a reference control for normalization. All primer sequences used in RT-qPCR detection are listed in Additional file 5: Table S2.

### Enzyme-linked immunosorbent assay (ELISA) and western blot (WB) analysis

CCL2 (SinoBiological, KIT10134), CCL20 (Abcam, ab269562), and CCL24 (Abcam, ab10050) levels in culture supernatant after treatment for 48 h were measured using ELISA kits. RIPA buffer with protease inhibitor and protein phosphatase inhibitor (APPLYGEN) was used for cell lysis. BCA reagent (APPLYGEN) was applied to detect the protein concentration. Cell lysates were loaded to 10% SDS-PAGE gel and transferred to PVDF membranes after separation. The following antibodies were used to detect proteins. GAPDH (CST, 14C10), VEGFA (Abcam, ab46154), STAT3 (CST, 79D7), p-STAT3 (Tyr705) (CST, D3A7), JAK2 (CST, D2E12), p-JAK2 (Tyr1007/1008) (CST, C80C3). Goat anti-rabbit IgG was used as the secondary antibody (CST).

### Gene knockdown by siRNA

We plated 2 × 10^5^/well H3122CR cells on 6-well plates for 24 h. Following the manufacturer’s instructions, cells were transfected with siRNA (Sangon Biotech) using Lipofectamine 3000 (Invitrogen). After transfection for 24–48 h, RT-qPCR or ELISA analysis was applied to verify the efficiency of gene knockdown. Cells transfected with nonsense siRNA duplexes were used as a control. Target sequences for siRNAs are listed in Additional file 5: Table S3.

### Cell growth and viability assay

3 × 10^3^/well cells were cultured for 0–72 h in 96-well plates to draw the cell growth curve. For dose–response curve, we plated cells in 96-well plates for 24 h and then treated with nine concentrations (0, 0.008, 0.04, 0.2, 1, 5, 10, 20, 40 μM) of crizotinib or anlotinib for 48 h. CCK-8 (Dojindo, Japan) assay was conducted to determine cell viability. Half maximal inhibitory concentration (IC50) values were calculated from the dose–response curves using GraphPad Prism 8 software.

### Cell cycle and apoptosis assays

2 × 10^5^ H3122CR cells were treated with siRNA after growing in 6-well plates for 24 h. To analyze the cell cycle phase, cells treated for 42 h were stained with propidium iodide (PI) (Dojindo, Japan). To evaluate cell apoptosis, Annexin V–fluorescein isothiocyanate (FITC) and PI were used to stain the cells after treatment for 48 h (Dojindo, Japan). Flow cytometry was used to analyze prepared samples (BD FACSCalibur).

### Colony formation assay

H3122CR cells (500 cells per well) were cultured in 6-well plates for 24 h and then exposed to 1 μM anlotinib for 2 weeks. Due to the weaker colony-forming ability of H3122 and H2228 cells, H3122 and H2228 (4000 cells per well) were cultured in 6-well plates for 48 h and then exposed to 1 μM anlotinib for 10 days. 100% methanol and 0.1% crystal violet were used to fix and stain the cell colonies for 20 min, respectively.

### Tube formation assay

Cell culture medium (CM) was collected after treatment for 24 h. 2 × 10^4^ HUVECs (human umbilical vein endothelial cells) were suspended in 50 μl CM and planted on 96-well plates coated with 50 μl Matrigel (Corning, USA). The number of tubes and capillary length were assessed after seeding for 4 h using ImageJ software.

### Animal experiments

Four-week-old female BALB/c nude mice were acquired from HFK Bioscience (Beijing, China) and maintained under specific pathogen-free conditions. To construct the H3122CR-derived xenograft model, 5 × 10^6^ H3122CR cells were subcutaneously injected into the right flank of mice. When the tumor volume reached to 100 mm^3^, mice were randomized to four groups and treated daily (day 0) by oral gavage as follows: (a) Control group (n = 6): 0.2% CMC-Na; (b) Crizotinib monotherapy group (n = 6): crizotinib (50 mg/kg/d) alone; (c) Anlotinib monotherapy group (n = 6): anlotinib (3 mg/kg/d) alone for two consecutive weeks and then discontinued for one week; (d) Combination treatment (n = 6): crizotinib (50 mg/kg/d) combined with anlotinib (3 mg/kg/d) for two consecutive weeks and then crizotinib alone for one week. Body weight and tumor volume were measured every two or three days, and tumor volume was calculated from the following formula: tumor volume (mm^3^) = length × width^2/2. At the end of the experiment (day 26), mice were sacrificed and tumors were collected and weighed. All animal experiments were approved by the Animal Care and Use Committee of Cancer Hospital, Chinese Academy of Medical Sciences (No. NCC2018A026).

### Analysis of The Cancer Genome Atlas (TCGA) and Gene Expression Omnibus (GEO) cohort

mRNA expression levels and clinical data for 497 lung adenocarcinoma (LUAD) patients were downloaded from TCGA dataset (The Pan-Cancer Atlas, http://www.cbioportal.org, https://gdc.cancer.gov/about-data/publications/pancanatlas). According to the median value of mRNA expression level, patients were divided into high expression group and low expression group to compare the disease-free survival (DFS) and OS. The comparison analysis of mRNA expression between lung squamous carcinoma (LUSC), LUAD, and normal tissue, and the correlation analysis between two genes were performed according to the Gene Expression Profiling Interactive Analysis (GEPIA, http://gepia.cancer-pku.cn/). GSE94089 dataset was obtained from GEO database containing RNA-seq data of 4 cell lines, including H2228, H2228-crizotinib resistance (CRR), H2228-ceritinib resistance (CER), and H2228-alectinib resistance (ALR). GEO2R was used to screen the DEGs between H2228 and H2228-resistance cell lines. The thresholds for significant DEGs selection were p-value < 0.05 and log2^Fold Change^ > 1. Then 381 significant DEGs were used to perform GO enrichment analysis using DAVID Bioinformatics Resources 6.8 and Cytoscape v3.8.2 software (https://david.ncifcrf.gov/, https://cytoscape.org/).

### Statistical analysis

In this study, data are presented as mean ± SD. Log-rank test was performed in univariable analysis using Kaplan–Meier survival analysis. COX regression model was conducted in multivariable analysis. R package “limma” was used for differential expression chemokines analysis. Three independent experiments of biological replicates were performed in all cell experiments excluding RNAseq. Student’s t-test was conducted in continuous variables. And Chi-square test was performed in categorical variables. SPSS 25.0, GraphPad Prism 8, and R packages were utilized for statistical analysis.

## Results

### Patient characteristics

A total of 61 *EML4-ALK* positive NSCLC patients receiving crizotinib were enrolled in the current study. The median age was 49 years old, with 40 (65.6%) patients were female. Pre-treatment plasma samples were collected from all 61 patients, and samples after disease progression were also collected from 15 cases with PFS > 12 months. The median follow-up time was 52.4 (IQR: 38.0–82.43) months. The detailed patient characteristics are listed in Table 1.

### Plasma chemokines levels reflect and predict crizotinib response in *EML4-ALK* positive NSCLC patients.

**Fig. 1:**
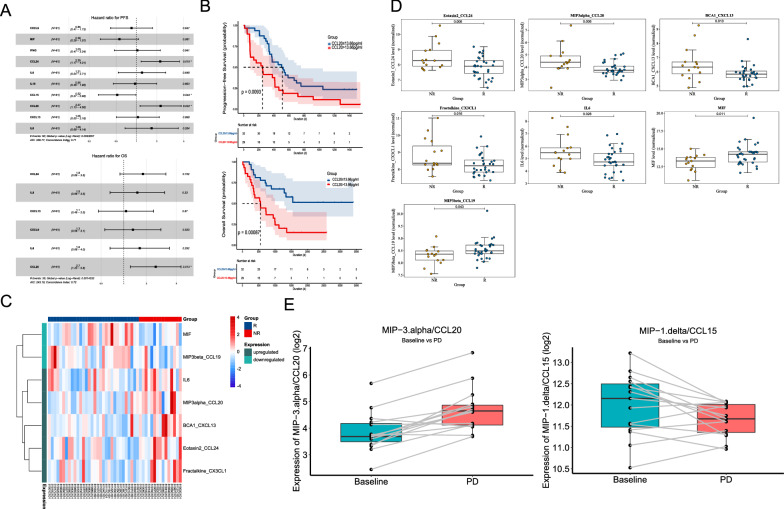
Plasma chemokines reflect and predict crizotinib response in *EML4-ALK* positive non-small cell lung cancer patients. **A** Forest plots of multivariant COX analysis results for progression-free survival (PFS) and overall survival (OS); **B** Kaplan–Meier curves of PFS and OS for CCL20 concentration; **C** Heatmap of 7 significant chemokines between patients with PFS shorter than 6 months (NR, n = 32) and longer than 12 months (R, n = 15); **D** Box plots of 7 significant chemokines expression comparing responders (R, n = 15) and non-responders (NR, n = 32); **E** Plasma CCL20 and CCL15 levels in baseline samples and samples after disease progression (PD)

To investigate the correlation between plasma chemokines expression levels and crizotinib efficacy, the baseline expression levels of 40 chemokines were measured in 61 *EML4-ALK* positive NSCLC patients before crizotinib treatment using the Luminex liquid suspension chip. The median value of each chemokine was used as a cut-off value for distinguishing the high expression and low expression groups. Univariate analysis suggested that 10 chemokines levels were significantly associated with PFS (CCL15, MIF, CCL20, CCL24, CXCL9, CXCL13, IFNG, IL6, IL8, and IL10), and 6 of which were also related to OS (CCL20, CCL24, CXCL9, CXCL13, IL6, and IL8) (Table 2). For most significant chemokines, high baseline levels indicated a poor prognosis for crizotinib treatment, only CCL15 and MIF were correlated with superior response. Then all these significant chemokines were included in multivariate Cox hazard analysis. Results showed that only 3 chemokines (CCL24, CCL15, and CCL20) remained to be associated with PFS and CCL20 was the independent factor for OS (Table 2; Fig. 1A). Overall, high expression of CCL20 and CCL24 and low expression of CCL15 were significantly correlated with poor clinical outcomes. The Kaplan–Meier curve for CCL20 was shown in Fig. 1B, with other significant chemokines in Additional file 1: Figure S1, Additional file 2: Figure S2. Moreover, time-dependent receiver operating characteristic curve (ROC) for CCL20 showed that the area under curve (AUC) was 0.78 for progression-free survival (PFS) and 0.76 for overall survival (OS) at 5 years (Additional file 2: Figure S2F, G). The clinical characteristics of patients with high CCL20 concentration and low CCL20 concentration were shown in Additional file 5: Table S4.

**Table 2:** Univariate and multivariate Cox hazard analysis of risk factors for clinical outcomes of *ALK* + NSCLC patients

	Univariate analysis			Multivariate analysis		
	HR	95%CI	p value	HR	95%CI	p value
PFS						
CXCL9	1.8	1–3.1	0.045	0.85	0.41–1.7	0.65
MIF	0.56	0.32–0.98	0.043	0.56	0.29–1.1	0.081
IFNgamma	1.8	1–3.2	0.041	1	0.47–2.2	0.94
CCL24	1.8	1–3.2	0.039	2.2	1.2–4.3	0.015
IL6	1.9	1.1–3.3	0.029	1.2	0.54–2.7	0.65
IL10	1.9	1.1–3.4	0.029	0.95	0.46–2	0.89
CCL15	0.53	0.3–0.93	0.029	0.52	0.27–0.98	0.044
CCL20	2.1	1.2–3.6	0.011	2.3	1.1–4.6	0.022
CXCL13	2.1	1.2–3.7	0.0084	1	0.5–2.2	0.9
IL8	2.2	1.2–3.9	0.0071	1.7	0.68–4.1	0.26
OS						
CCL24	2	1–4	0.043	1.8	0.89–3.6	0.1
IL8	2.2	1.1–4.4	0.022	1.5	0.66–3.5	0.33
CXCL13	2.7	1.3–5.4	0.0059	1.1	0.46–2.5	0.87
CXCL9	2.6	1.3–5.2	0.0053	1.3	0.56–3.1	0.52
IL6	2.7	1.3–5.3	0.005	1.6	0.66–4	0.29
CCL20	3.1	1.5–6.1	0.0015	2.7	1.2–5.8	0.013

*ALK* anaplastic lymphoma kinase, *NSCLC* non-small cell lung cancer, *PFS* progression-free survival, *HR* hazard ratio, *CI* confidence interval, *OS* overall survival

Subsequently, we divided patients with PFS longer than 12 months and PFS shorter than 6 months into two groups, which were defined as responders and non-responders, respectively, to screen the differential expression chemokines for crizotinib primary resistance. Overall, most significant chemokines were elevated in non-responders’ baseline plasma samples (Fig. 1C). CCL20, CCL24, and other 3 chemokines (CXCL13, IL6, and CX3CL1) significantly increased in non-responders, while MIF and CCL19 were highly expressed in responders (Fig. 1D). Remarkably, CX3CL1 and CCL19 did not show a significant difference in the whole cohort.

To further explore the association between chemokines expression and acquired resistance to crizotinib, the dynamic changes of chemokines in paired baseline plasma samples and progression samples were analyzed. Among 15 responders with PFS > 12 months, plasma CCL20 level was low at baseline and remarkably elevated after crizotinib resistance, while CCL15 level significantly decreased upon resistance (Fig. 1E). Collectively, these results suggested that CCL20 possibly plays a crucial role in crizotinib resistance.

### The functional enrichment and TCGA cohort analysis of chemokines related to crizotinib response.

We next sought to explore the underlying molecular mechanisms of resistance via interaction molecules of the significant chemokines related to crizotinib efficacy found above. The interaction molecules of 12 chemokines (CCL15, MIF, CCL20, CCL24, CXCL9, CXCL13, IFNG, IL6, IL8, IL10, CX3CL1, and CCL19) were downloaded from the BioGRID database (https://thebiogrid.org/, Additional file 5: Table S5). A total of 266 interactors were used for functional enrichment analysis. The results suggested that multiple biological processes (BPs) were involved in crizotinib resistance, such as numerous BPs related to chemokines, leukocyte chemotaxis, and migration (Additional file 3: Figure S3A). Interestingly, we found that several angiogenesis-related BPs (including positive regulation of angiogenesis and vasculature development, tube development, blood vessel development, and vasculature development) were enriched among interaction molecules of 12 chemokines (Additional file 3: Figure S3B).

We then analyzed the expression of 12 significant chemokines in NSCLC tissue from the TCGA and GEPIA database. GEPIA results revealed that the mRNA expression of CCL15 was significantly downregulated in LUSC and LUAD compared with normal lung tissues, while CCL20, CXCL9, CXCL13 were upregulated in tumor tissues (Additional file 3: Figure S3C), demonstrating that high expression of CCL20 may lead to tumorigenesis, while CCL15 behaved as a protective factor. Next, we downloaded a dataset from the TCGA (n = 497) to explore the association between significant chemokines and clinical outcomes in LUAD. We found that high CCL20 mRNA expression in tumor tissues significantly indicated shorter DFS and OS for LUAD patients (Additional file 3: Figure S3D), which further confirmed that CCL20 can be used to monitor and predict clinical outcomes in LUAD patients.

### Transcriptome analysis indicates that high mRNA expression of chemokines is involved in crizotinib resistance.

**Fig. 2:**
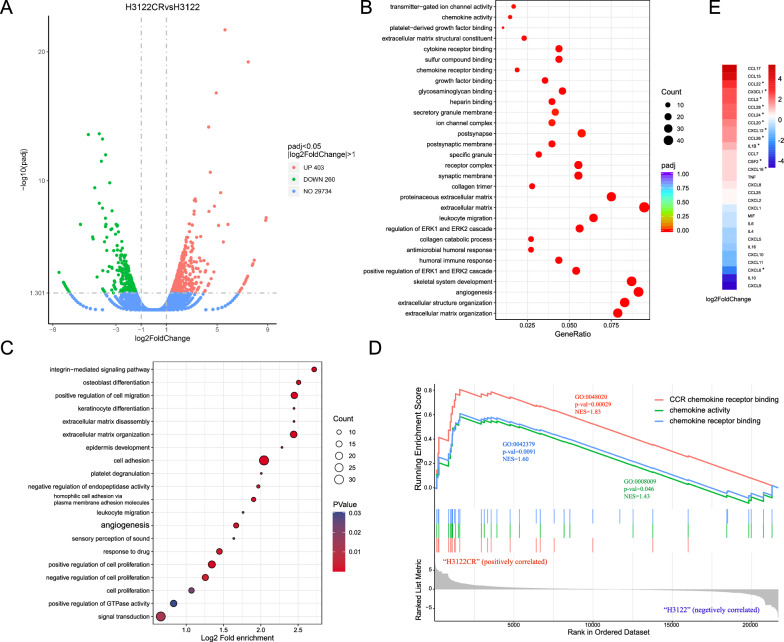
Transcriptome analysis indicates that chemokines overexpression and angiogenesis are involved in crizotinib resistance. **A** Volcano plot of all differentially expressed genes (DEGs) between H3122 and H3122CR; **B** Top 30 Gene Oncology (GO) terms enriched among DEGs between H3122 and H3122CR; **C** Top 20 biological processes of DEGs from GSE94089; **D** Gene Set Enrichment Analysis (GSEA) of DEGs between H3122 and H3122CR; **E** The fold change of mRNA expression of chemokines in H3122CR compared to H3122

To confirm the correlation between chemokines and crizotinib resistance, RNA-seq was performed to identify the DEGs and enriched biological processes between H3122 and H3122CR cell lines. 403 upregulated DEGs and 260 downregulated DEGs were obtained in H3122CR after differential expression analysis (Fig. 2A). First, we identified biological processes enriched among DEGs by GO enrichment analysis. The results suggested that extracellular matrix, angiogenesis, leukocyte migration, regulation of ERK1 and ERK2 cascade, chemokine receptor binding, and chemokine activity were involved in crizotinib resistance (Fig. 2B). Interestingly, biological processes related to angiogenesis were also enriched in DEGs from another independent GEO dataset (GSE94089) with an expression profile of H2228 and ALK-TKI-resistant (crizotinib, ceritinib, and alectinib) H2228 cell lines (Fig. 2C, Additional file 4: Figure S4A).

Next, all expression dataset from H3122 and H3122CR cell lines was included in the GSEA analysis. Results showed that three chemokine-related molecular functions (CCR chemokine receptor binding, chemokine activity, and chemokine receptor binding) were positively correlated with H3122CR (Fig. 2D). We then compared the mRNA expression of 40 chemokines detected in plasma samples between H3122 and H3122CR and found that 3 chemokines (CCL20, CCL24, and CX3CL1) significantly overexpressed in H3122CR compared with H3122. It was consistent with the trend in clinical samples (Fig. 2E). In some ways, high expression of chemokines and angiogenesis pathways may contribute to ALK-TKI resistance in *ALK*-positive NSCLC.

### CCL20 and CCL24 are the key factors leading to crizotinib resistance.

**Fig. 3:**
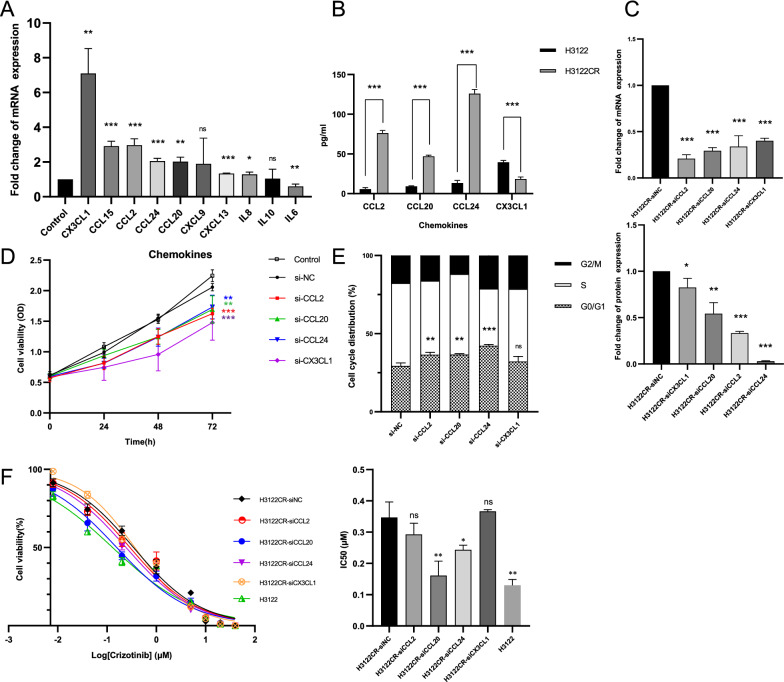
CCL20 and CCL24 involved in crizotinib resistance. **A** 10 chemokines mRNA expression in H3122CR compared with H3122 (Control); **B** 4 chemokines protein expression in H3122 and H3122CR cell supernatants; **C** The mRNA and protein expression of 4 chemokines after si-RNA treatment compared with si-negative control (si-NC); **D–F** Effect of CCL2, CCL20, CCL24, and CX3CL1 knockdown on (D) cell proliferation of H3122CR; (E) cell cycle distribution of H3122CR; **F** half maximal inhibitory concentration (IC50) of crizotinib in H3122CR. * p < 0.05, ** p < 0.01, *** p < 0.001, ns: not significant

qPCR was applied to verify the RNA-seq results of clinical-significant chemokines in H3122 and H3122CR. qPCR showed that CCL20, CCL24, and CX3CL1 were significantly up-regulated in H3122CR (Fig. 3A). But the high expression of CCL15 in crizotinib-resistant cells was not consistent with the clinical results. Since CCL2 plays a crucial role in angiogenesis and was highly expressed in H3122CR, we focused on these four chemokines (CCL2, CCL20, CCL24, CX3CL1) in subsequent studies [19]. ELISA and liquid chips were adopted to detect protein levels of these four chemokines in cell culture supernatant. Except for CX3CL1, the protein levels of the other three chemokines were consistent with mRNA expression (Fig. 3B).

To investigate the impact of 4 chemokines on H3122CR cell line, we suppressed CCL2, CCL20, CCL24, and CX3CL1 expression in H3122CR by chemokine-specific siRNA, respectively. mRNA and protein detection showed that siRNA successfully reduced the expression of chemokines (Fig. 3C). Cell proliferation curve showed that si-CCL2, si-CCL20, si-CCL24, and si-CX3CL1 inhibited the viability of H3122CR compared to the blank (Control) and siRNA negative control (si-NC) (Fig. 3D). Cell cycle distribution suggested that cell proliferation arrested in G0/G1 phase after si-CCL2, si-CCL20, or si-CCL24 treatment, which may explain the proliferation inhibition phenomenon above (Fig. 3E). Moreover, the apoptosis assay demonstrated that si-CCL2, si-CCL20, si-CCL24, and si-CX3CL1 did not increase the incidence of apoptosis (Additional file 4: Figure S4B).

To further confirm the impact of 4 chemokines on crizotinib resistance, IC50 of crizotinib was calculated in H3122CR after si-CCL2, si-CCL20, si-CCL24, and si-CX3CL1 treatment, respectively. Compared with si-NC, knockdown of CCL20 and CCL24 significantly reversed crizotinib resistance in H3122CR, while si-CCL2 and si-CX3CL1 treatment did not affect the sensitivity to crizotinib, which was consistent with the results in clinical samples (Fig. 3F). CX3CL1 only showed a clinical correlation in patients with PFS > 12 months and PFS < 6 months but not in the whole cohort, which may explain the inconsistency in cell experiments. Although CCL2 and CX3CL1 affected cell viability in some ways, these two chemokines may not be the key factors for crizotinib resistance. Combined with the clinical results, we suggest that high expression of CCL20 and CCL24 are the key factor involved in crizotinib resistance in vitro and in clinical samples.

### CCL20 may induce crizotinib resistance by activation of angiogenesis via JAK2/STAT3-CCL20-VEGFA/IL6 axis

**Fig. 4:**
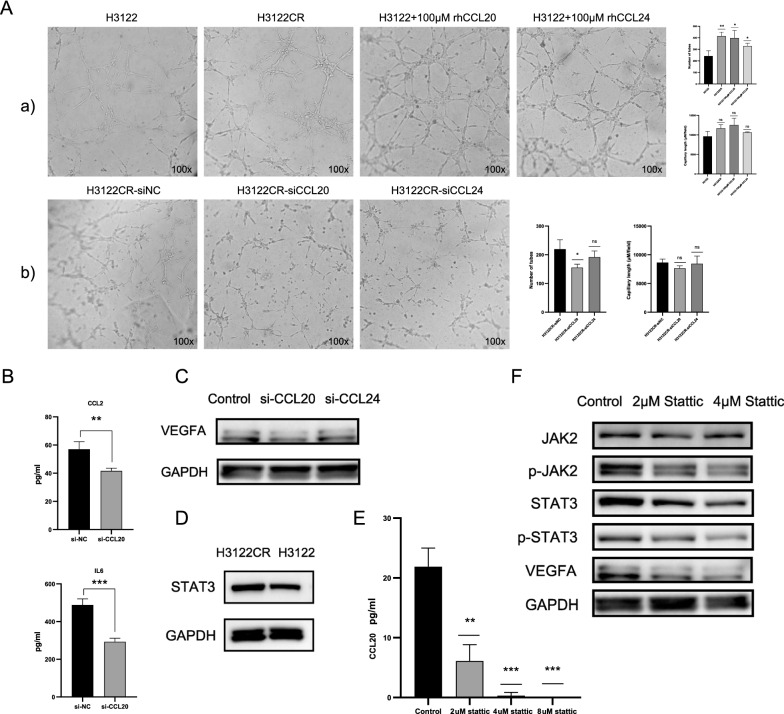
CCL20 may induce crizotinib resistance by activation of angiogenesis via JAK2/STAT3-CCL20-VEGFA/IL6 axis. **A** a) Tube formation of HUVECs cultured in H3122 culture medium (CM), H3122CR CM, H3122 CM supplemented with human recombinant CCL20, and H3122 CM supplemented with rhCCL24. b) Tube formation of HUVECs cultured in the CM of H3122CR transfected with si-negative control (si-NC), si-CCL20, and si-CCL24. The number of tubes and capillary length were analyzed to evaluate angiogenic activity of CCL20 and CCL24; **B**, **C** Knockdown of CCL20 suppressed the protein expression of CCL2, IL6, and VEGFA in H3122CR compared with si-NC (Control); **D** STAT3 was upregulated in H3122CR compared with H3122; **E**, **F** Stattic inhibited the JAK2/STAT3 pathway and the protein expression of CCL20 and VEGFA in H3122CR. GAPDH served as a loading control. *p < 0.05, **p < 0.01, ***p < 0.001, *ns* not significant

Angiogenesis is highly induced during the growth and progression of tumor [4]. Based on the observation of angiogenesis enrichment among DEGs in cell lines and clinical samples, we hypothesized that high expression of chemokines lead to crizotinib resistance by activation of angiogenesis pathways. Tube formation assay was performed to explore the angiogenic ability of CCL20 and CCL24. The results showed that HUVECs cultured with culture medium of H3122CR formed more tubes than H3122 (Fig. 4A). Adding human recombinant CCL20 and CCL24 in H3122 culture medium promoted the tube formation of HUVECs significantly, while the knockdown of CCL20 and CCL24 in H3122CR suppressed the tube formation of HUVECs (Fig. 4A). Thereinto, CCL20 has a more significant effect on inducing angiogenesis.

As CCL20 played crucial roles in crizotinib efficacy in vitro & vivo and was involved in the prognosis of LUAD patients from the TCGA cohort, the molecular mechanisms of CCL20 activating angiogenesis were further explored. Interestingly, GEPIA correlation analysis indicated that CCL20 was positively correlated with CCL24 in LUAD, which may explain the consistent impact of two chemokines on crizotinib sensitivity and verify the key role of CCL20 in crizotinib resistance (Additional file 4: Figure S4C). Moreover, angiogenesis-related genes including VEGFA, IL6 and CCL2 were positively correlated with the mRNA expression of CCL20 (Spearman’s q = 0.2, 0.27, and 0.15; all p-values are < 0.01, Additional file 4: Figure S4C). After si-CCL20 treatment, the protein levels of IL6 and CCL2 in cell supernatant were markedly decreased, and WB analysis showed that VEGFA was downregulated after CCL20 knockdown in H3122CR (Fig. 4B, C). Previous studies have reported that JAK2/STAT3 pathway regulated the expression of CCL20 and tumor angiogenesis [20, 21]. In this study, we found that STAT3 was upregulated in H3122CR compared with H3122 cells (Fig. 4D). To investigate whether STAT3 affects the expression of CCL20 in H3122CR cells, we inhibited STAT3 phosphorylation in H3122CR by stattic treatment. Results indicated that the expression of CCL20 and VEGFA were declined after stattic treatment (Fig. 4E, F). Based on these observations, we speculated that CCL20 induced crizotinib resistance by the activation of angiogenesis via JAK2/STAT3-CCL20-VEGFA/IL6 axis.

### Anlotinib reverses crizotinib resistance by inhibiting the chemokines-induced angiogenesis

**Fig. 5:**
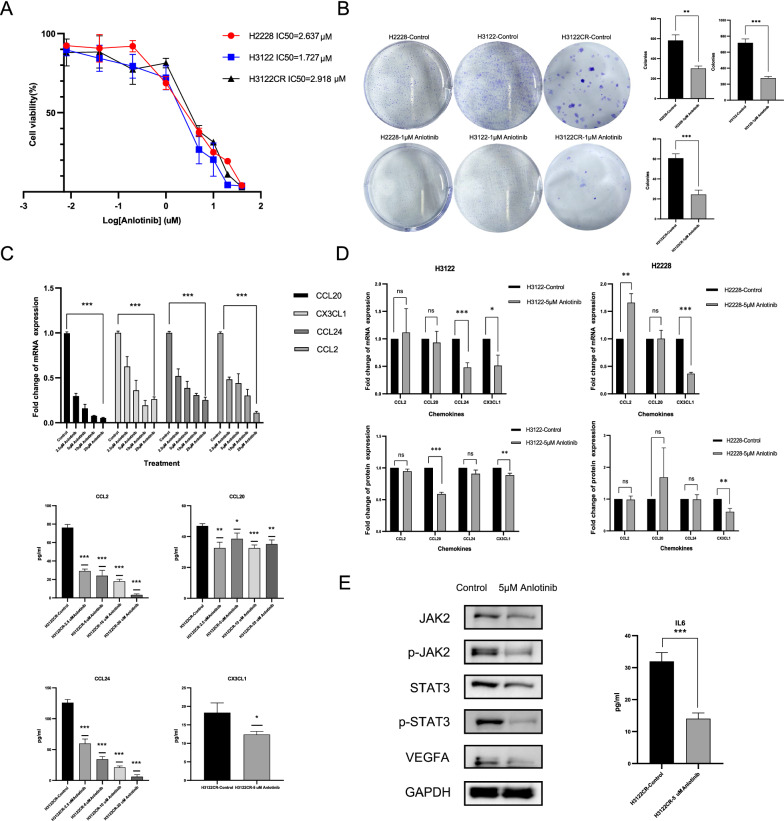
The inhibition effect of anlotinib on *ALK*-positive cell lines. **A** Dose response curve and half maximal inhibitory concentration (IC50) of anlotinib in H2228, H3122, and H3122CR; **B** Effect of anlotinib treatment on cell colony formation in H2228, H3122, and H3122CR; **C** Anlotinib inhibited the mRNA and protein expression of CCL20, CCL24, CCL2, and CX3CL1 in H3122CR; **D** Effect of anlotinib treatment on chemokines expression in H3122 and H2228; **E** Anlotinib inhibited the JAK2/STAT3-VEGFA/IL6 axis. GAPDH served as a loading control. * p < 0.05, ** p < 0.01, *** p < 0.001, ns: not significant

**Fig. 6:**
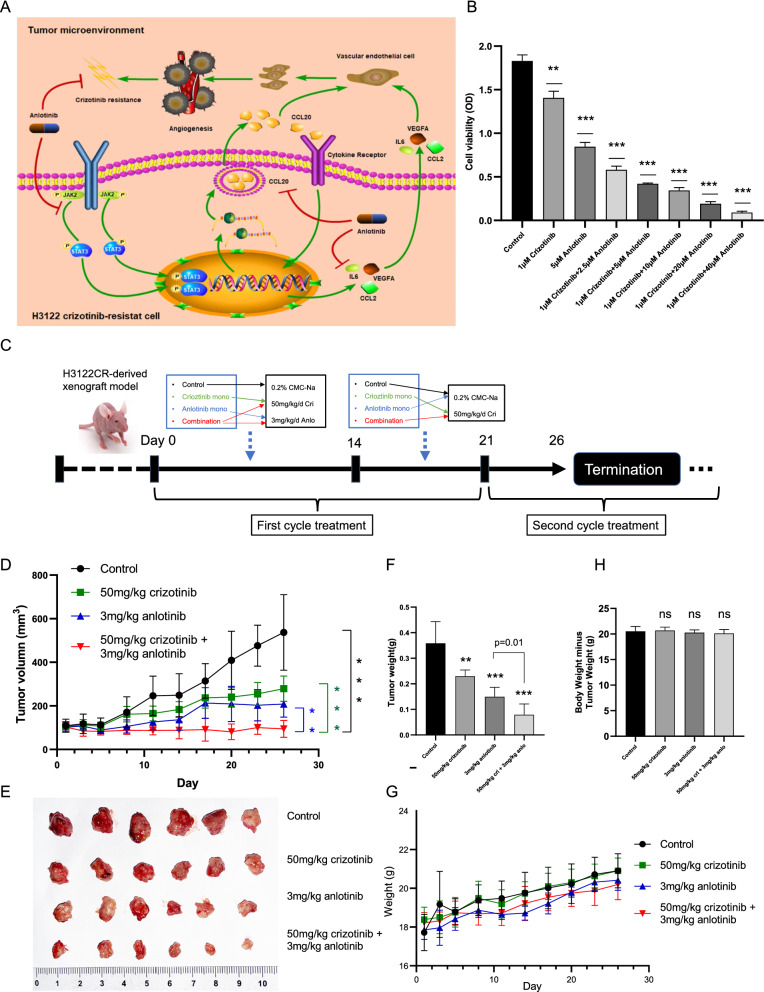
Antitumor acitivity of crizotinib combined with anlotinib in H3122CR-derived xenograft model. **A** Schematic diagram of anlotinib reversing crizotinib resistance by inhibition of angiogenesis via JAK2/STAT3-CCL20-VEGFA/IL6 axis; **B** Inhibition effect of anlotinib combined with crizotinib on H3122CR compared with anlotinib or crizotinib monotherapy; **C** Timeline of the treatment in mice; **D** Measurements of subcutaneous tumor volumes in the control, crizotinib monotherapy, anlotinib monotherapy, and crizotinib combined with anlotinib groups (n = 24); **E** Tumors were obtained after mice were sacrificed on Day 26; **F** Measurement of tumor weight at the end of the experiment; **G** Body weight growth curves of nude mice during the treatment; **H** Body weight minus tumor weight was analyzed at the end of the experiment. *p < 0.05, **p < 0.01, ***p < 0.001, *ns* not significant

Previous studies have indicated that anti-angiogenic TKI anlotinib inhibits tumor growth by decreasing chemokine expression [19]. Here, we asked whether anlotinib could overcome crizotinib resistance by inhibiting chemokines-induced angiogenesis. Dose–response curve and colony formation assay showed the cytotoxicity induced by anlotinib in H2228, H3122, and H3122CR (Fig. 5A, B). The mRNA and protein expression of CCL20, CCL24, CCL2, and CX3CL1 were decreased in H3122CR after anlotinib treatment, respectively (Fig. 5C). However, the changes of these four chemokines expression in H3122 and H2228 after anlotinib treatment were not as marked as H3122CR, which indicated that the inhibitory effect of anlotinib on chemokines is specific to H3122CR (Fig. 5D). Moreover, WB results showed that anlotinib inhibited the expression of JAK2, STAT3, and VEGFA (Fig. 5E). IL6 in cell supernatant was also declined after anlotinib treatment (Fig. 5E). The schematic diagram of anlotinib reversing crizotinib resistance by inhibition of angiogenesis via JAK2/STAT3-CCL20-VEGFA/IL6 axis is shown in Fig. 6A.

Given the cytotoxicity induced by anlotinib in crizotinib-resistant cells, we next investigated whether the combination of crizotinib and anlotinib could enhance the ability to reverse crizotinib resistance. Surprisingly, in vitro experiments showed that the combination of two drugs was more effective than monotherapy (Fig. 6B). Then, to examine whether anlotinib combined with crizotinib has a better antitumor effect than monotherapy in vivo, we administered the combination drugs in H3122CR-derived xenograft models. The schematic diagram of the treatment is shown in Fig. 6C. Results showed that the combination treatment significantly improved the antineoplastic ability compared to crizotinib or anlotinib monotherapy (p < 0.05, Fig. 6D–F). Moreover, the mice had no obvious toxic or side effects after the combined treatment, and their body weight gradually increased during the treatment cycle (Fig. 6G). No significant difference was observed in body weight between the four treatment groups (p > 0.05, Fig. 6H). Results in in vivo experiments were consistent with our previous conclusions in vitro, which indicated that the combination of anlotinib with crizotinib is a promising treatment for patients resistant to crizotinib with acceptable toxicity.

## Discussion

Angiogenesis leads to tumor occurrence, progression, and drug resistance [4, 5, 22]. Here, we reported that chemokine-induced angiogenesis drives resistance to crizotinib in *ALK*-positive patients. This discovery has provided possible plasma chemokine biomarkers for response prediction in ALK-TKIs treated patients and guided us to explore the therapeutic effect of anlotinib monotherapy and combination therapy in ALK-TKIs resistant patients.

Although researches on prognostic markers for chemotherapy and immunotherapy have increased dramatically in NSCLC patients, biomarkers for ALK-targeted therapy are rarely reported. One of the clear evidence is that mutated *TP53* correlated to unfavorable crizotinib PFS in *ALK*-positive patients [23, 24]. Another prognostic marker for ALK-TKI therapy is *ALK* variant type, but it remains contentious. Yoshida, et al. firstly found that *EML4-ALK* variant 1 was correlated with superior prognosis versus non-v1, while Woo, et al. demonstrated that patients with v3a/b had shorter PFS after crizotinib treatment [25, 26]. However, some other studies indicated no significant correlation between *ALK* fusion variants and clinical outcomes [27]. Due to the difficulty of tissue sample collection, circulating tumor cells and cell-free DNA in plasma samples were also investigated to monitor the duration and magnitude of clinical response of patients receiving ALK-TKIs [28, 29]. In the current study, we showed that 3 chemokines (CCL24, CCL15, and CCL20) in baseline plasma samples were associated with PFS, and baseline CCL20 was an independent prognostic factor for OS in patients treated with crizotinib. Subsequently, we reported that baseline CCL20 and CCL24 were significantly elevated in patients with PFS < 6 months compared with patients with PFS > 12 months, indicating that CCL20 and CCL24 possibly contributed to the primary resistance to crizotinib. Furthermore, the detection of baseline and progression samples demonstrated that the dynamic changes of CCL20 and CCL15 can monitor the acquired resistance to crizotinib. TCGA and GEPIA datasets also verified that high expression of CCL20 and low expression of CCL15 were significantly related to the initiation of LUAD. Due to the convenience of plasma chemokines detection, CCL20, CCL24, and CCL15 can serve as prognostic markers to identify crizotinib resistance and predict the clinical outcomes in *ALK*-positive patients. However, we failed to confirm the molecular function of CCL15 in cell experiments. Contrary to clinical results, CCL15 overexpressed in H3122CR instead of H3122 cells. Previous studies mainly reported that CCL15 was involved in the occurrence and development of hepatocellular carcinoma (HCC) [30]. Highly expression of CCL15 was correlated with dismal survival in HCC patients [30]. But the effect of CCL15 on lung cancer remains controversial. One previous study indicated that the CCL15 level significantly decreased when patients had a partial response after erlotinib and celecoxib treatment [31]. However, according to our study, CCL15 decreased when patients acquired drug resistance and progressed. The complexity of the effect of CCL15 on NSCLC may explain the inconsistent results in vivo & vitro experiments in the current study.

Based on the observation of angiogenesis enriched both in clinical samples and cell lines, we explored the role of angiogenesis in the process of CCL20-induced resistance. We demonstrated that CCL20 boosted angiogenesis via JAK2/STAT3-CCL20-VEGFA/IL6 axis to confer resistance to crizotinib. Subsequently, anti-angiogenic drug anlotinib was applied to overcome resistance, showing an inhibitory effect on 4 chemokines and JAK2/STAT3-VEGFA/IL6 axis. It suggested that anlotinib not only suppressed angiogenesis in the tumor microenvironment but also inhibited angiogenesis pathways in tumor cells. Similarly, a previous study found the consistent phenomenon that anlotinib suppressed JAK2/STAT3/VEGFA pathway in NSCLC xenograft tumors [32]. Moreover, Lu, et al. indicated that anlotinib inhibited tumor growth in an *EGFR*-mutant NSCLC xenograft model by restraining the CCL2-induced angiogenesis, which further confirmed our results [19]. Interestingly, the suppression effect of anlotinib on these 4 chemokines was not significant in crizotinib-sensitive H3122 and H2228 cells. This is possible because chemokines dramatically overexpressed in H3122CR compared with H3122, and anlotinib showed a stronger inhibitory effect on chemokines to reverse drug resistance. The underlying mechanism of anlotinib inhibiting H3122 and H2228 needs further exploration.

Preclinical researches and clinical trials have demonstrated that anti-angiogenic drugs combined with EGFR-TKIs is a viable strategy for *EGFR*-mutant NSCLC patients [33–36]. According to the results of an ongoing phase II clinical trial, impressive objective response rate and disease control rate were obtained with acceptable toxicity after the united medication [33]. Li, et al. indicated that the combination of anlotinib with gefitinib enhanced the inhibition to cell proliferation in vitro and tumor angiogenesis in xenograft models [35]. Here, based on the inhibitory effect of anlotinib on H3122CR cells and H3122CR-derived xenografts, we firstly reported a combination strategy of anlotinib with crizotinib for crizotinib-resistant *ALK*-positive NSCLC. Results showed that anlotinib boosted the anti-tumor effect of crizotinib in vitro & in vivo. Similarly, a recent study has reported that the combination of anti-VEGFR2 antibody with crizotinib augmented the effect of anti-proliferative effects on tumor cells [36]. Moreover, a phase II clinical trial combining alectinb with bevacizumab in *ALK*-positive non-squamous NSCLC patients with alectinib resistance showed clinical efficacy and acceptable toxicity in vivo [37]. Therefore, the combination therapy of anlotinib with ALK-TKIs could serve as a promising treatment strategy for *ALK*-positive NSCLC patients.

Several limitations should be noted in our study. One limitation is the small sample size of the included patient cohort. And due to the limited sample size of *EML4-ALK* fusion NSCLC in TCGA dataset, we only validated the prognostic efficacy of CCL20 in LUAD patients but not in *ALK*-positive patients. Besides, we have found several chemokines related to crizotinib response in clinical samples but only the underlying mechanism of CCL20 was clarified in this study. Thirdly, clinical trials will be carried out to verify the anti-tumor effect of anlotinib monotherapy and combination therapy on *ALK*-positive patients in the future. Moreover, we mainly focused on the treatment strategies for ALK-TKIs-resistant NSCLC in the current study. Hence, the efficacy and molecular mechanism of anlotinib alone or combined with ALK-TKIs for the untreated *ALK*-positive patients still need further exploration.

## Conclusion

Together, CCL20-mediated angiogenesis is involved in crizotinib resistance and could be overcome by anlotinib in *EML4-ALK* positive NSCLC. Plasma CCL20 can serve as an efficacy predictive biomarker for *ALK*-positive patients receiving crizotinib treatment. The combination of anlotinib with crizotinib is a promising treatment for patients resistant to ALK-TKIs.

## Supplementary Information

**Additional file 1: Figure S1**. Kaplan-Meier survival analysis of 9 significant chemokines with progression-free survival (PFS). (A) CCL15; (B) MIF; (C) CCL24; (D) CXCL9; (E) CXCL13; (F) IFN-gamma; (G) IL-6; (H) IL-8; (I) IL-10.

**Additional file 2: Figure S2**. Kaplan-Meier survival analysis of 5 significant chemokines with overall survival (OS) and time-dependent operating characteristic curve (ROC) of CCL20. (A) IL-8; (B) CCL24; (C) CXCL9; (D) CXCL13; (E) IL-6; Time-dependent operating ROC of CCL20 (F) for progression-free survival (PFS); (G) for OS.

**Additional file 3: Figure S3**. The functional enrichment and TCGA cohort analysis of chemokines related to crizotinib efficacy. (A) Biological processes (BPs) enriched among the interaction molecules of 12 chemokines; (B) Angiogenesis-related BPs enriched among the interaction molecules by Cytoscape; (C) The mRNA expression of 4 chemokines between lung adenocarcinoma (LUAD), lung squamous carcinoma (LUSC), and normal tissue from the GEPIA database; (D) Kaplan-Meier curves of disease-free survival (DFS) and overall survival (OS) for CCL20 mRNA expression in TCGA cohort. * p < 0.05.

**Additional file 4: Figure S4**. The functional enrichment and GEPIA analysis of significant genes and the results of cell apoptosis assay. (A) Angiogenesis-related BPs enriched among the differentially expressed genes from GSE94089 dataset by Cytoscape; (B) Effect of si-CCL2, si-CCL20, si-CCL24, and si-CX3CL1 treatments on cell apoptosis in H3122CR; (C) GEPIA correlation analysis for CCL20 with VEGFA, IL6, CCL2, and CCL24. ns: not significant.

**Additional file 5: Table S1**. 40 chemokines included in the Bio-Plex Pro Human Chemokine Panel. **Table S2**. The primer sequences used for RT-qPCR detection. **Table S3**. Target sequences for siRNAs. **Table S4**. Clinical characteristics of patients with different CCL20 expression. **Table S5**. The interaction molecules of 12 chemokines downloaded from BioGRID database

## Data Availability

All data that support the findings of this study are available from the corresponding author upon reasonable request.

## Abbreviations

EML4-ALK: Echinoderm microtubule associated protein like 4-anaplastic lymphoma kinase
NSCLC: Non-small cell lung cancer
TKIs: Tyrosine kinase inhibitors
IC50: Half maximal inhibitory concentration
VEGFA: Vascular endothelial growth factor-A
H3122CR: H3122-crizotinib resistant cell
PFS: Progression-free survival
OS: Overall survival
EGFR: Epidermal growth factor receptor
HUVEC: Human umbilical vein endothelial cells
DEGs: Differentially expressed genes
RNA-seq: RNA sequencing
FPKM: Fragments per kilobase per million
GO: Gene ontology
GSEA: Gene set enrichment analysis
RT-PCR: Reverse transcription polymerase chain reaction
qPCR: Quantitative real-time polymerase chain reaction
ELISA: Enzyme-linked immunosorbent assay
WB: Western blot
CM: Cell culture medium
LUAD: Lung adenocarcinoma
DFS: Disease-free survival
LUSC: Lung squamous carcinoma
HCC: Hepatocellular carcinoma
