# Supplementary figures and images for: Role of chemokine-mediated angiogenesis in resistance towards crizotinib and its reversal by anlotinib in EML4-ALK positive NSCLC
Source: J Transl Med. 2022 May 31;20:248. doi: 10.1186/s12967-022-03451-2 (PMC9153090; doi:10.1186/s12967-022-03451-2)

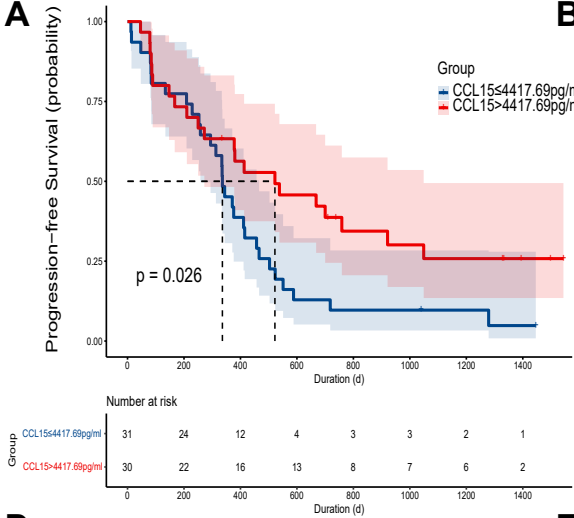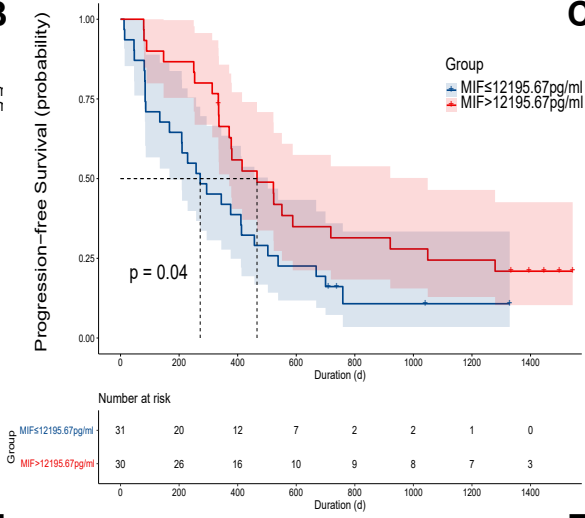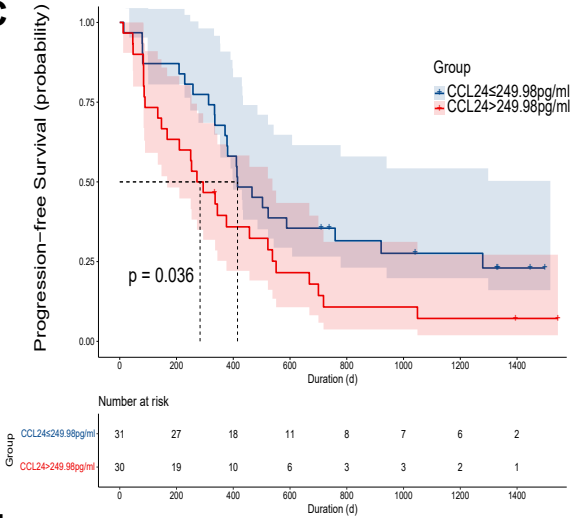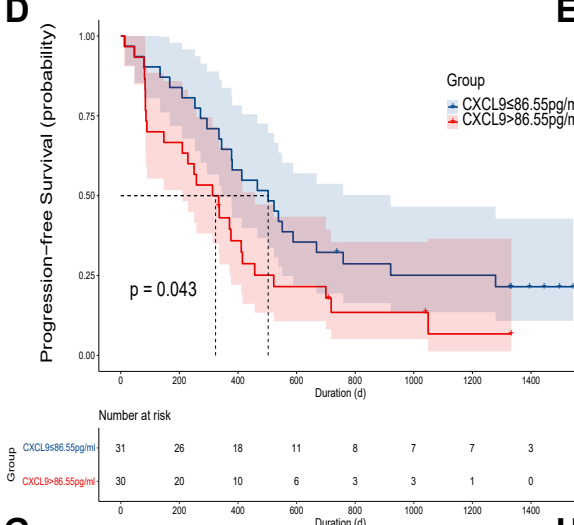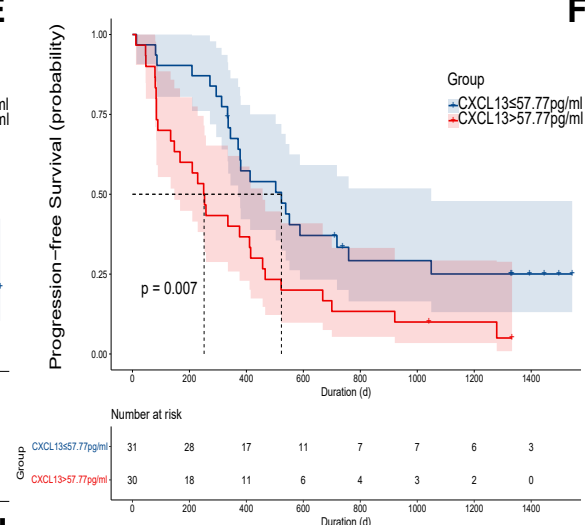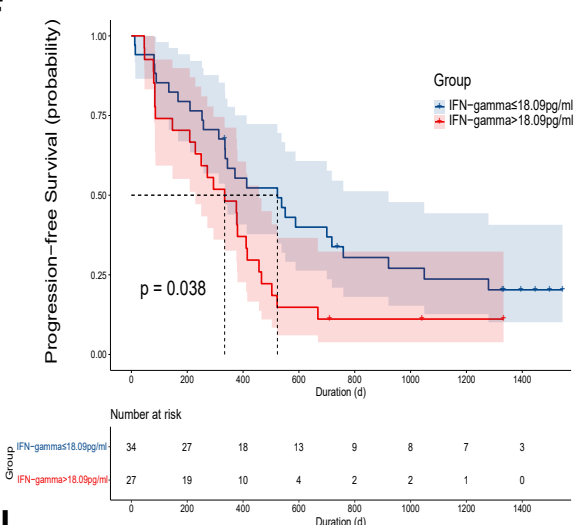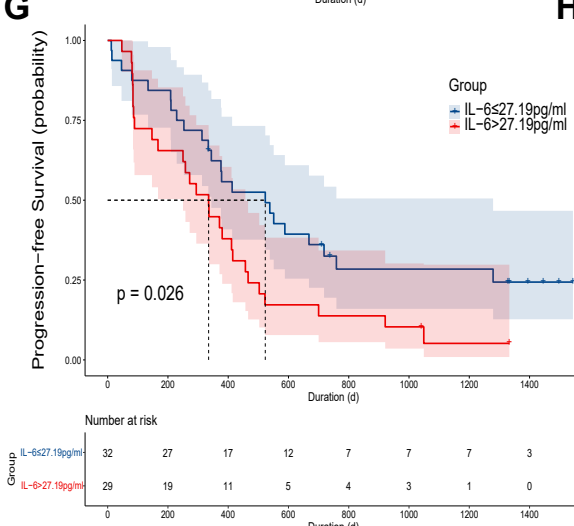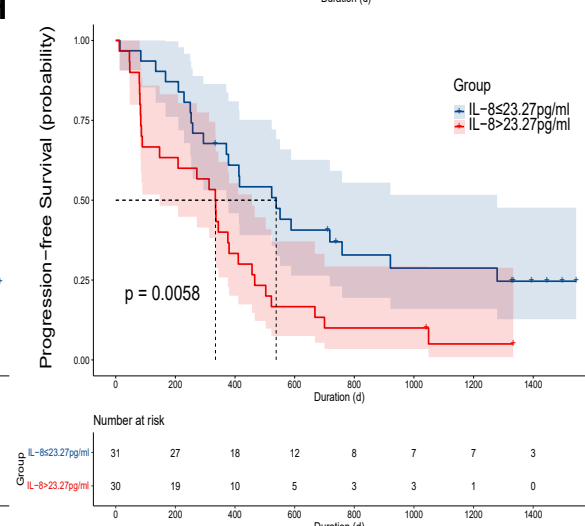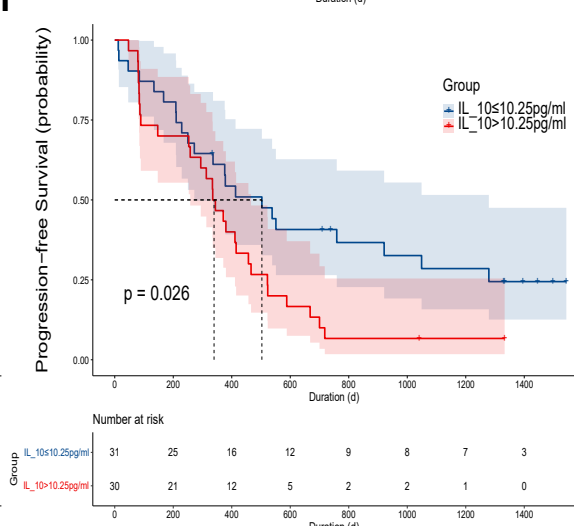

Supplement: Supplementary file 1 — Additional file 1: Figure S1. Kaplan-Meier survival analysis of 9 significant chemokines with progression-free survival (PFS). (A) CCL15; (B) MIF; (C) CCL24; (D) CXCL9; (E) CXCL13; (F) IFN-gamma; (G) IL-6; (H) IL-8; (I) IL-10. [file 12967_2022_3451_MOESM1_ESM.pdf]

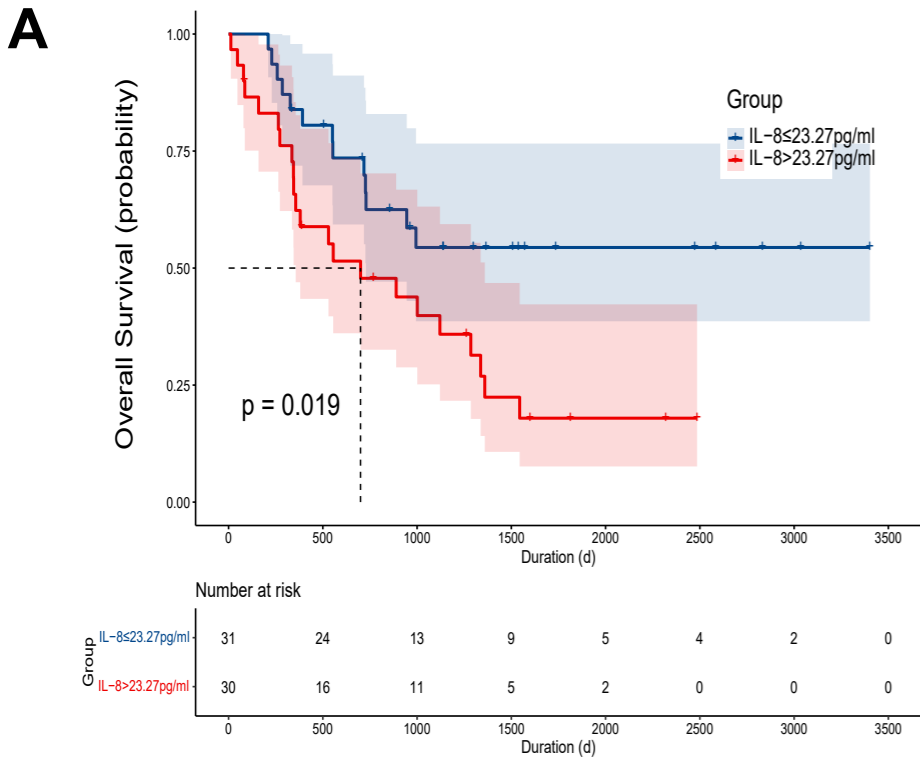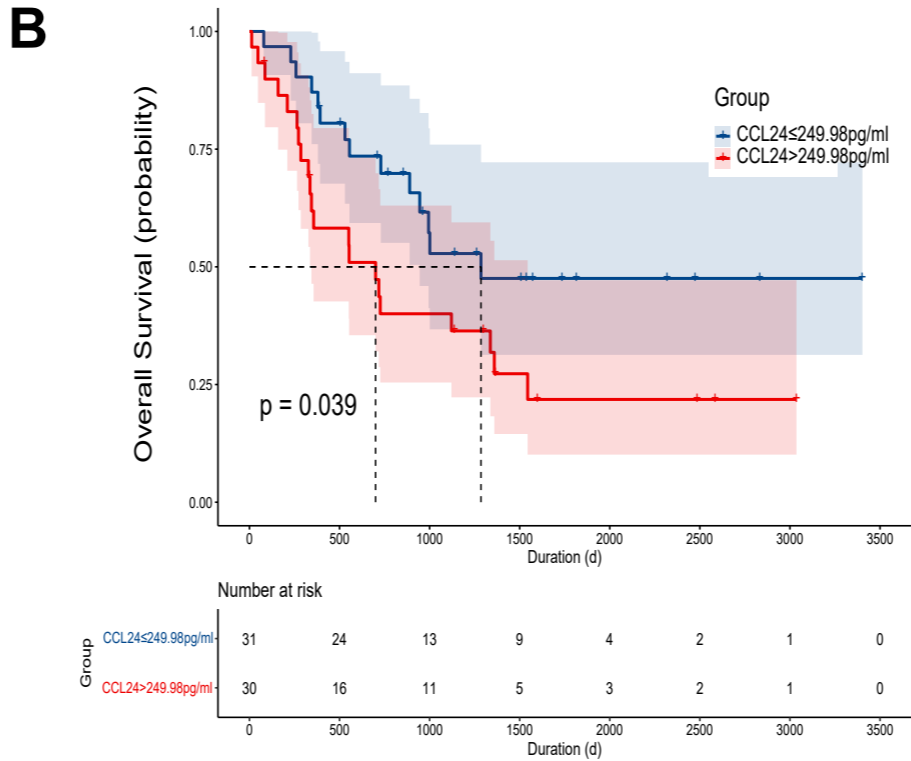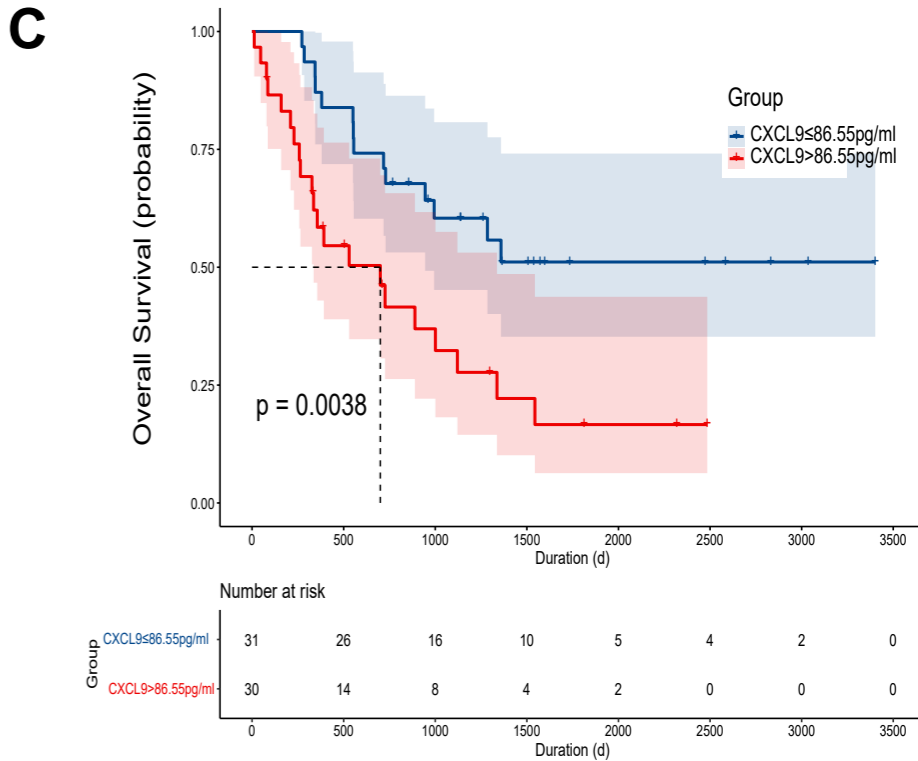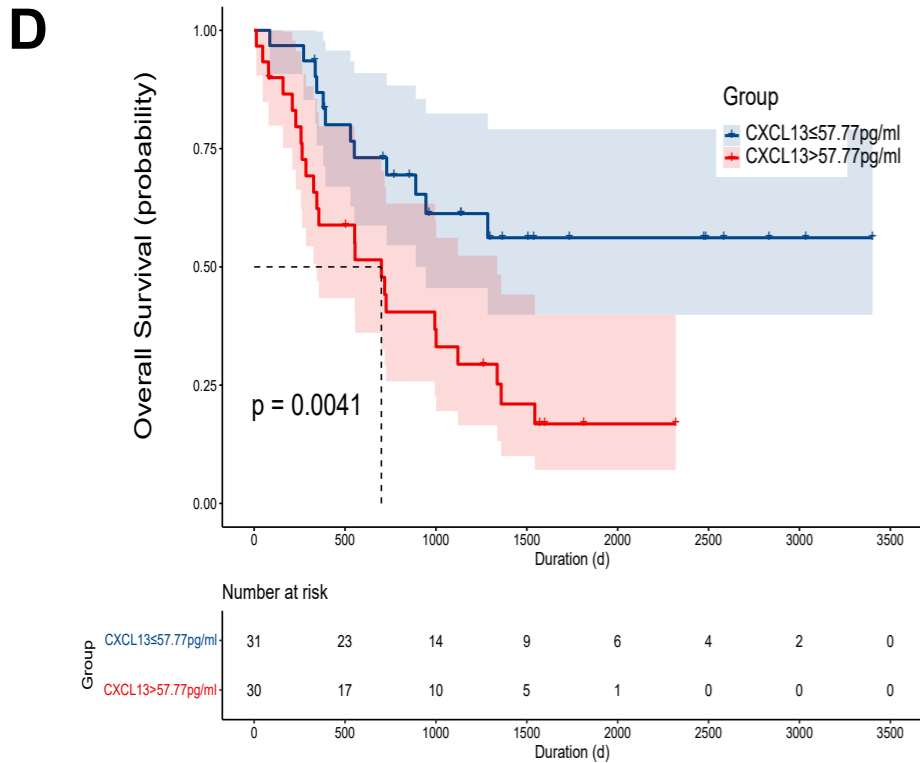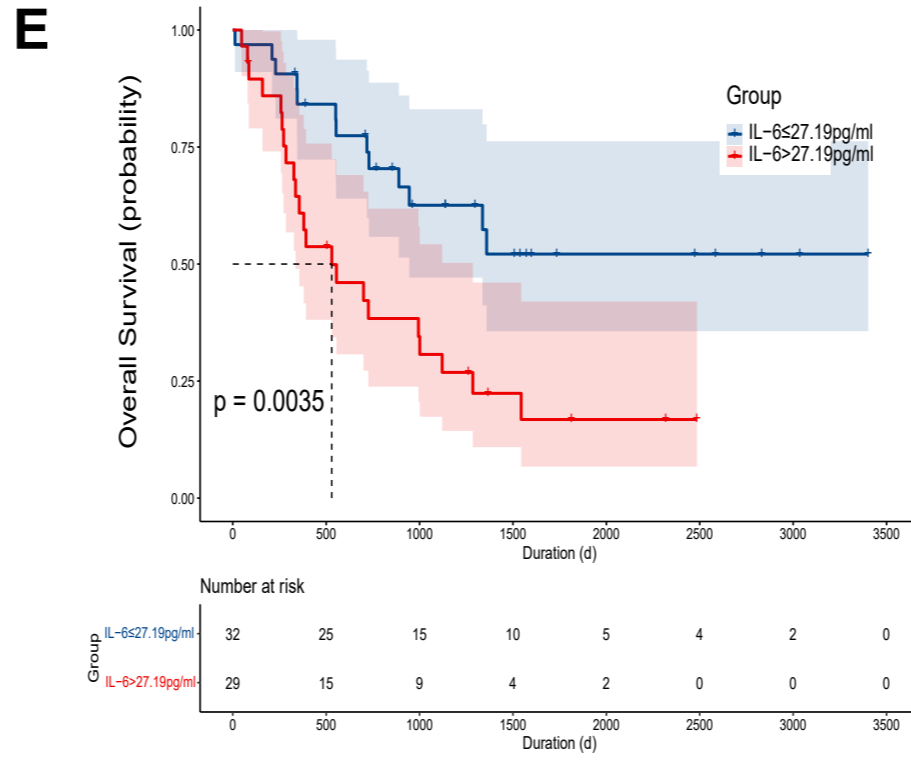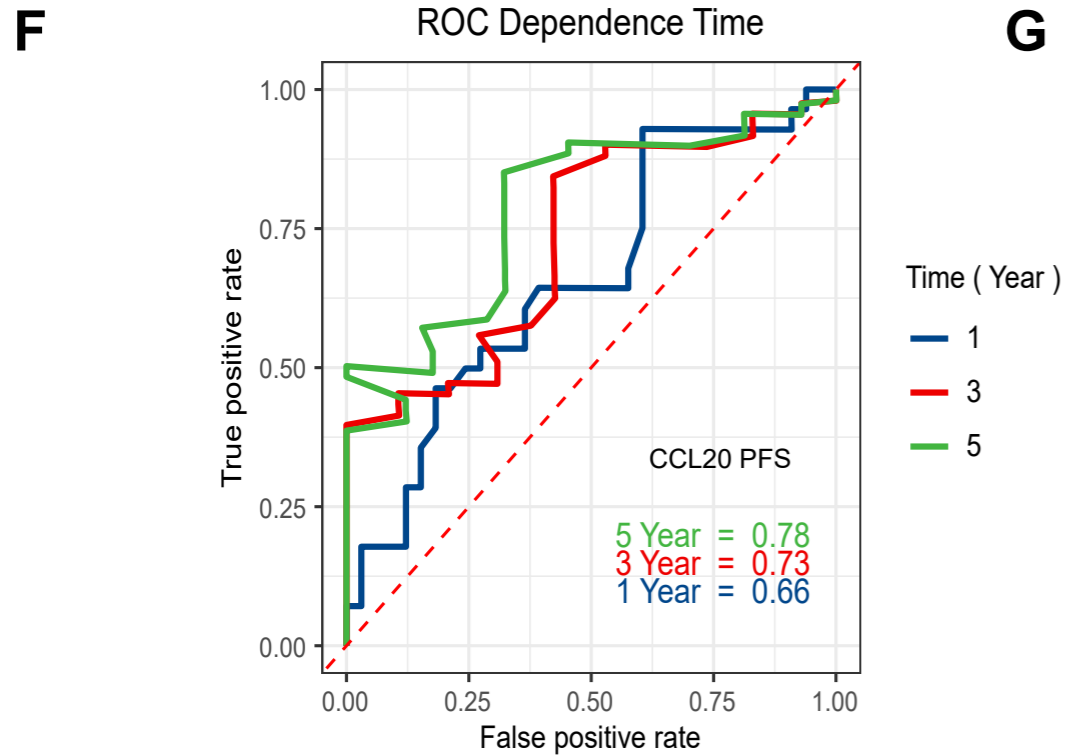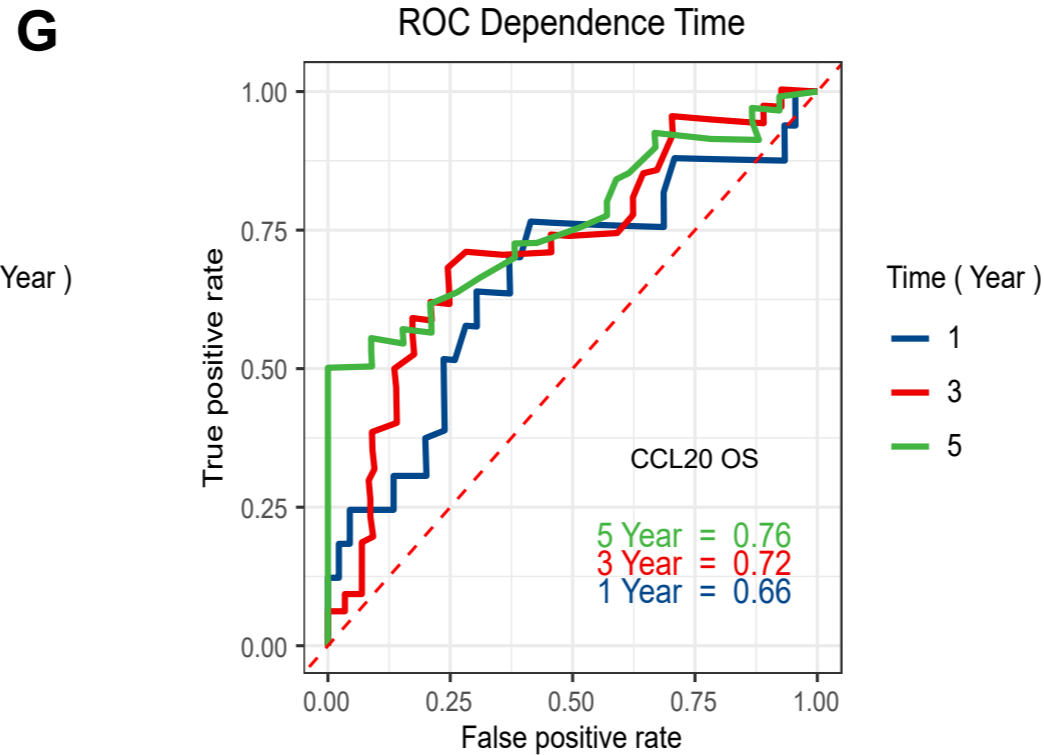

Supplement: Supplementary file 2 — Additional file 2: Figure S2. Kaplan-Meier survival analysis of 5 significant chemokines with overall survival (OS) and time-dependent operating characteristic curve (ROC) of CCL20. (A) IL-8; (B) CCL24; (C) CXCL9; (D) CXCL13; (E) IL-6; Time-dependent operating ROC of CCL20 (F) for progression-free survival (PFS); (G) for OS. [file 12967_2022_3451_MOESM2_ESM.pdf]

A

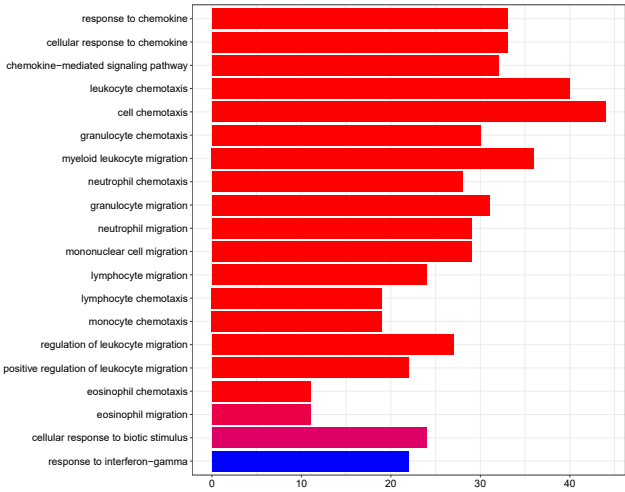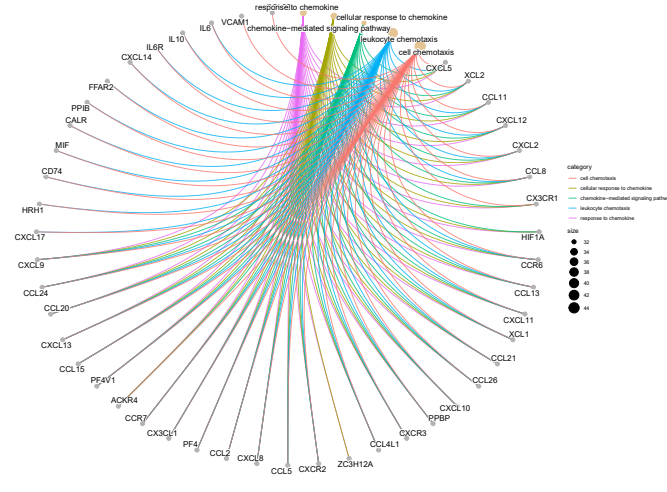

B

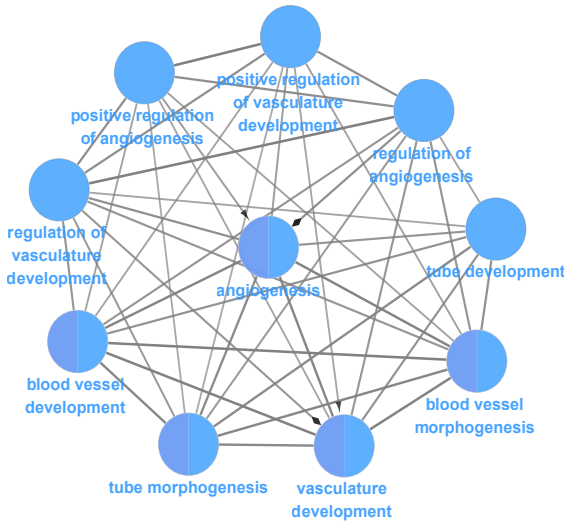

C

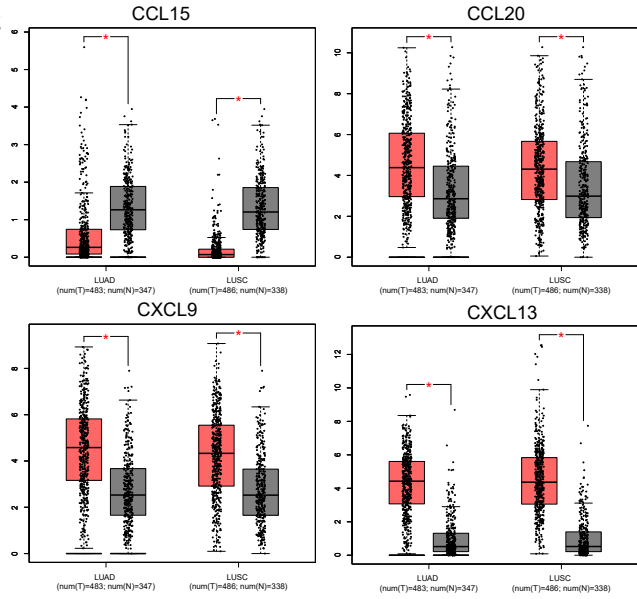

D

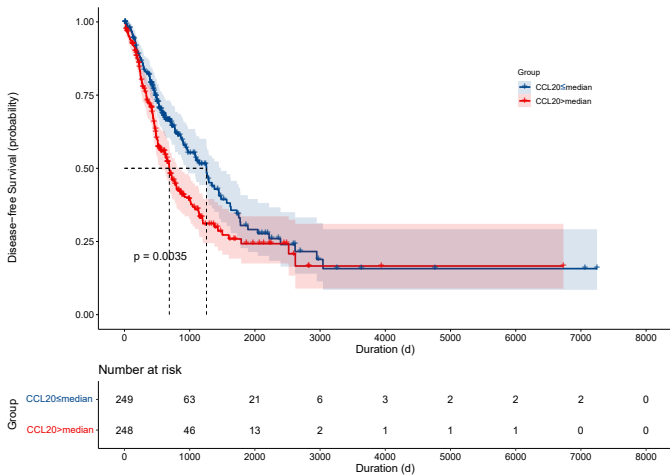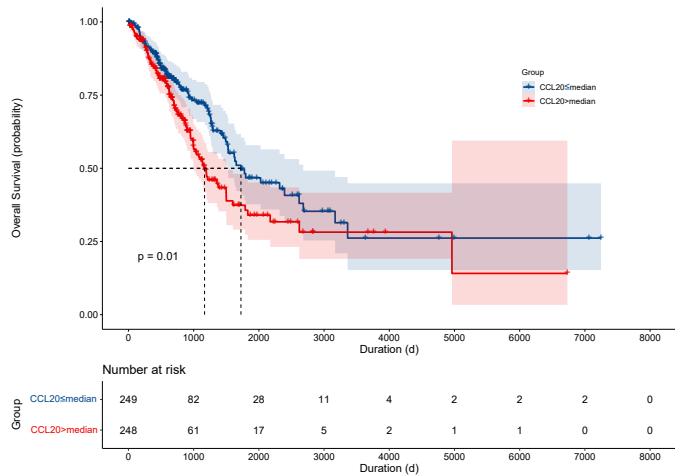

Supplement: Supplementary file 3 — Additional file 3: Figure S3. The functional enrichment and TCGA cohort analysis of chemokines related to crizotinib efficacy. (A) Biological processes (BPs) enriched among the interaction molecules of 12 chemokines; (B) Angiogenesis-related BPs enriched among the interaction molecules by Cytoscape; (C) The mRNA expression of 4 chemokines between lung adenocarcinoma (LUAD), lung squamous carcinoma (LUSC), and normal tissue from the GEPIA database; (D) Kaplan-Meier curves of disease-free survival (DFS) and overall survival (OS) for CCL20 mRNA expression in TCGA cohort. * p < 0.05. [file 12967_2022_3451_MOESM3_ESM.pdf]

A

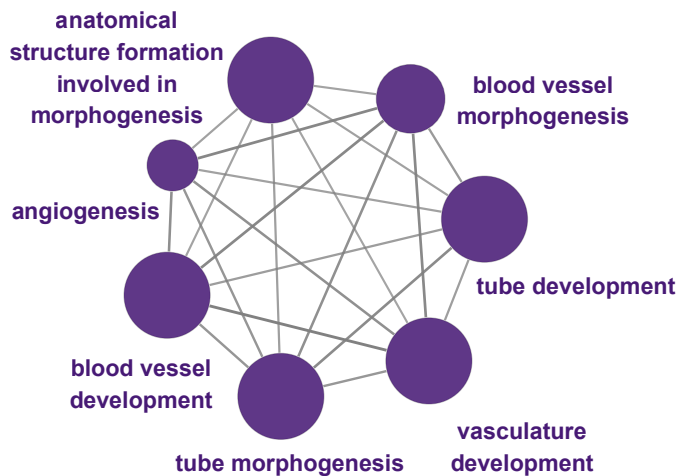

C

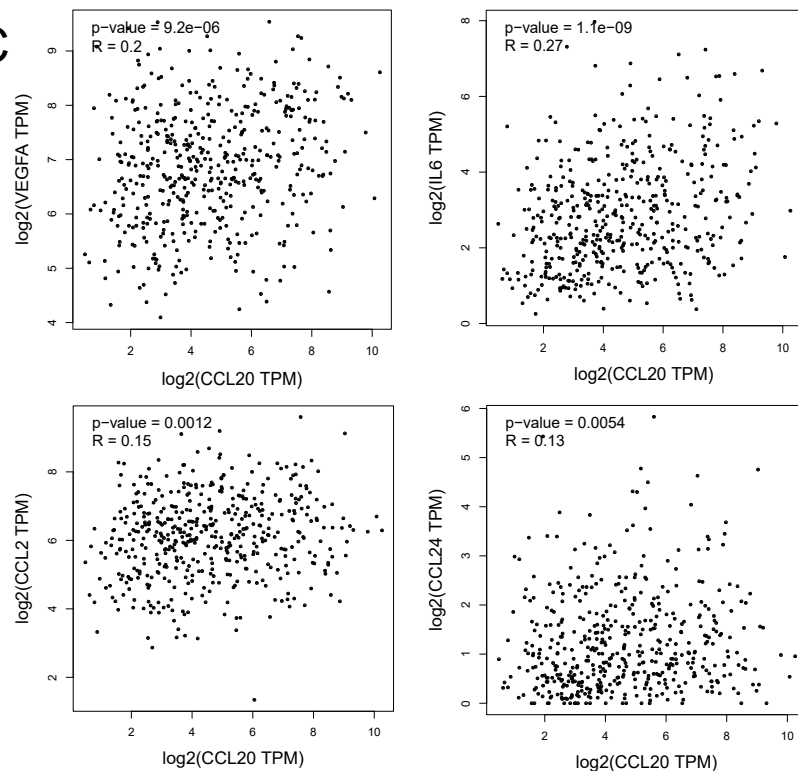

B

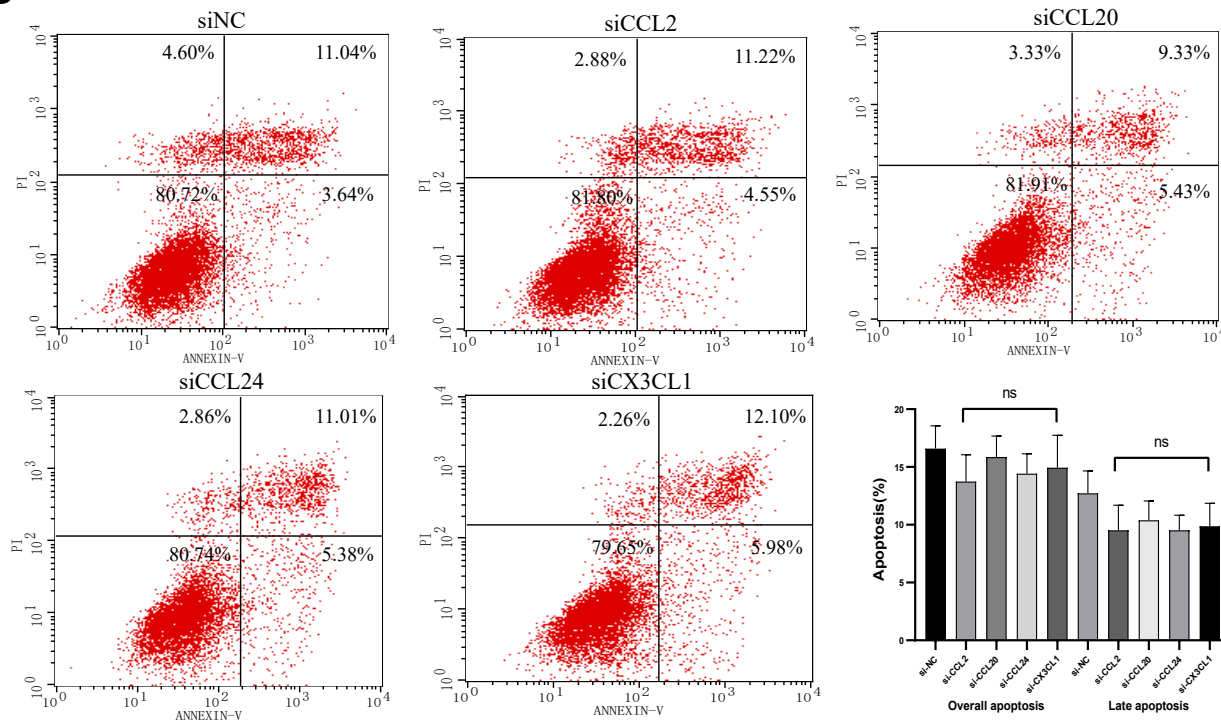

Supplement: Supplementary file 4 — Additional file 4: Figure S4. The functional enrichment and GEPIA analysis of significant genes and the results of cell apoptosis assay. (A) Angiogenesis-related BPs enriched among the differentially expressed genes from GSE94089 dataset by Cytoscape; (B) Effect of si-CCL2, si-CCL20, si-CCL24, and si-CX3CL1 treatments on cell apoptosis in H3122CR; (C) GEPIA correlation analysis for CCL20 with VEGFA, IL6, CCL2, and CCL24. ns: not significant. [file 12967_2022_3451_MOESM4_ESM.pdf]
