# Supplementary material for: Role of chemokine-mediated angiogenesis in resistance towards crizotinib and its reversal by anlotinib in EML4-ALK positive NSCLC
Source: J Transl Med. 2022 May 31;20:248. doi: 10.1186/s12967-022-03451-2 (PMC9153090; doi:10.1186/s12967-022-03451-2)
Supplement: Supplementary file 5 — Additional file 5: Table S1. 40 chemokines included in the Bio-Plex Pro Human Chemokine Panel. Table S2. The primer sequences used for RT-qPCR detection. Table S3. Target sequences for siRNAs. Table S4. Clinical characteristics of patients with different CCL20 expression. Table S5. The interaction molecules of 12 chemokines downloaded from BioGRID database [file 12967_2022_3451_MOESM5_ESM.docx]

**Table S1**

**40 chemokines included in the Bio-Plex Pro Human Chemokine Panel**

| 6Ckine/CCL21 | Fractalkine/CX3CL1 | IL-1β | IP-10/CXCL10 | MIF | SCYB16/CXCL16 |
| --- | --- | --- | --- | --- | --- |
| BCA-1/CXCL13 | GCP-2/CXCL6 | IL-2 | I-TAC/CXCL11 | MIG/CXCL9 | SDF-1α+β/CXCL12 |
| CTACK/CCL27 | GM-CSF | IL-4 | MCP-1/CCL2 | MIP-1α/CCL3 | TARC/CCL17 |
| ENA-78/CXCL5 | Gro-α/CXCL1 | IL-6 | MCP-2/CCL8 | MIP-1δ/CCL15 | TECK/CCL25 |
| Eotaxin/CCL11 | Gro-β/CXCL2 | IL-8/CXCL8 | MCP-3/CCL7 | MIP-3α/CCL20 | TNF-α |
| Eotaxin-2/CCL24 | I-309/CCL1 | IL-10 | MCP-4/CCL13 | MIP-3β/CCL19 |  |
| Eotaxin-3/CCL26 | IFN-γ | IL-16 | MDC/CCL22 | MPIF-1/CCL23 |  |

**Table S2**

**The primer sequences used for RT-qPCR detection**

| Gene | Forward 5'-3' sequences | Reverse 5'-3' sequences |
| --- | --- | --- |
| GAPDH | CTGGGCTACACTGAGCACC | AAGTGGTCGTTGAGGGCAATG |
| CX3CL1 | ACCACGGTGTGACGAAATG | TGTTGATAGTGGATGAGCAAAGC |
| CCL15 | TCCCAGGCCCAGTTCATAAAT | TGCTTTGTGAGATGTAGGAGGT |
| CCL2 | CAGCCAGATGCAATCAATGCC | TGGAATCCTGAACCCACTTCT |
| CCL24 | ACATCATCCCTACGGGCTCT | CTTGGGGTCGCCACAGAAC |
| CCL20 | TGCTGTACCAAGAGTTTGCTC | CGCACACAGACAACTTTTTCTTT |
| CXCL9 | AAGACCTTAAACAATTTGCCCC | TGCTGAATCTGGGTTTAGACAT |
| CXCL13 | GCTTGAGGTGTAGATGTGTCC | CCCACGGGGCAAGATTTGAA |
| IL8 | ACTGAGAGTGATTGAGAGTGGAC | AACCCTCTGCACCCAGTTTTC |
| IL10 | GACTTTAAGGGTTACCTGGGTTG | TCACATGCGCCTTGATGTCTG |
| IL6 | ACTCACCTCTTCAGAACGAATTG | CCATCTTTGGAAGGTTCAGGTTG |

RT-qPCR: reverse transcription-quantitative real-time polymerase chain reaction

**Table S3**

**Target sequences for siRNAs**

| siRNA | sense（5'-3'） | antisense（5'-3'） |
| --- | --- | --- |
| si-NC | UUC UCC GAA CGU GUC ACG UTT | ACG UGA CAC GUU CGG AGA ATT |
| si-CCL2 | CCCAGUCACCUGCUGUUAUAATT | UUAUAACAGCAGGUGACUGGGTT |
| si-CC20 | CGAAUCAGAAGCAGCAAGCAATT | UUGCUUGCUGCUUCUGAUUCGTT |
| si-CCL24 | GUUCUUUGUUUCCAAGAGAAUTT | AUUCUCUUGGAAACAAAGAACTT |
| si-CX3CL1 | GCUGCUGCCCUAACUCGAAAUTT | AUUUCGAGUUAGGGCAGCAGCTT |

**Table S4**

**Clinical characteristics of patients with different CCL20 expression.**

|  | CCL20-high | CCL20-low | p value |
| --- | --- | --- | --- |
| Sex |  |  | 0.583 |
| Female | 18 | 22 |  |
| Male | 11 | 10 |  |
| Age, median (SD) | 47 (13.0) | 50.5 (12.9) | 0.277 |
| Smoke |  |  | 0.945 |
| Yes | 5 | 6 |  |
| No | 14 | 16 |  |
| Chemotherapy |  |  | 0.377 |
| Yes | 23 | 29 |  |
| No | 6 | 3 |  |
| Brain Metastasis |  |  | 0.187 |
| Yes | 12 | 21 |  |
| No | 9 | 7 |  |
| Bone Metastasis |  |  | 0.297 |
| Yes | 16 | 15 |  |
| No | 9 | 15 |  |

SD: standard deviation.

**Table S5**

**The interaction molecules of 12 chemokines downloaded from BioGRID database**

| Chemokines | Interaction molecules |
| --- | --- |
| CCL15 | CCL2 |
| MIF | GORASP2,HSP90AA1,TP53,NGFRAP1,WDYHV1,UFM1,NEDD4,ANXA5,TPI1,PEBP1,MDH2,PDIA4,FIBP,CD74,VHL,STUB1,CDK2,NME1,IGBP1,CLNS1A,VCAM1,FN1,ITGA4,BNIPL,UPF2,ZNF408,ASB15,CUL7,AKR1C2,LOC101930400,ANXA11,CAPG,COX17,CRIP1,DSTN,ENO1,FSCN1,GBE1,HINT1,LAP3,LDHA,LDHB,MPI,NANP,PABPC1,PAICS,PCBP1,PCMT1,PDCD6,PDCD6IP,PIR,SPR,TIMM13,YKT6,CEP290,MKNK2,VPS26A,MCM2,EGFR,CDC73,DLD,GCD7,HIF1A,COPS5,PRPF8,EFTUD2,AAR2,PIH1D1,TNIP2,CHD4,SPDL1,ESR2,HEXIM1,MEPCE,LARP7,RECQL4,ZFP36L2,MYC,HIST1H4A,HIST1H2BB,KIAA1429,ATXN3,BIRC3,TEX101,LRRK2,MIF,ESR1,PINK1,YAP1,TFCP2,E7,HCVgp1,ZC3H18,S,ORF3a,M,E,ORF3b,CHMP4B,LDLR,INS,RBM45,Apc2,CALR,HSPE1,PPIB,PFN1,CALM1,PDIA3,STMN1,TRIM24,BASP1,SOD1,SUMO2,PTMA,PGAM1,ATPIF1,PTMS,ANP32B,PGK1,MARCKS,COPS7B,PARK7,HMGN1,TKT,PARK2,UFL1,DDRGK1,HCVgp1,PARK7, TKT, SOD1, TP53 |
| CCL20 | TGFB3,CCL5,CXCL11,CXCL12,CXCL17,PF4,CXCL5,XCL2,CCR6,VCAN,MTDH |
| CCL24 | CCL5,CXCL11,CCL13,CCL21,XCL1,DDX58 |
| CXCL9 | PAX8,PTPN5,CCDC155,SYNE4,RHCG,FCGR2A,SLC39A1,MR1,CHODL,TMEM237,SUSD3,TSPAN18,IFNGR2,TMEM234,TM4SF18,SSMEM1,CLDN2,BTNL9,CLEC4E,TMEM194A,C16orf92,BSCL2,SFTPC,GJB1,CLEC7A,FNDC9,LRRC25,TMEM31,LSMEM2,FUT7,AMIGO1,CLDN7,EPGN,TMPRSS2,CLDN5,MUC1,CLEC17A,LDLRAD1,LEUTX,KIR2DL3,CCL11,CCL13,CCL28,CCL5,CXCL11,CXCL12,CXCL17,CXCL2,XCL1,CXCR3,BMF,FBXO7,CLEC2D,AQP6,SLC10A1,ATP1B4,CD33,TNFSF8,TMED5,GORAB,MTIF3,EREG,TNFSF14,GPR42,FFAR2,TMEM106C,ITM2C,CCL2,CCL21,CCL26,CXCL10,CXCL14,PF4,PPBP |
| CXCL13 | EEF1G,CDKN1A,CXCR3,ACKR4,CCL28,CCL4L1,PF4V1 |
| IFNG | IFNGR2,STAT6,IFNGR1,GOPC,IFNG,DCAF4 |
| IL6 | SH3GL2,ZBTB16,HRH1,JAK1,IL6R,CSK,ZC3H12A,ZC3H12B,IL12RB1,RC3H1 |
| IL8 | SDC1,POMGNT1,SGTA,FANCI,CCL8,CXCR2,CCL5,tat,EP300,BAG6,ARF,AP3,TP53,CXCL8,ERBB2,CCL2,PF4,ZNF598,ZFP36 |
| IL10 | KRTAP10-8,KRTAP4-1,KRTAP1-3,NOTCH2NL,NBPF19,NFKB2,SPHK1, MCL1,IL10RA,A2M,IL10,YY1,GLRX3 |
| CX3CL1 | CX3CR1,CX3CL1 |
| CCL19 | CCR7,ACKR4,MEOX2,PF4V1 |

**Figure legends**

**Figure S1. Kaplan-Meier survival analysis of 9 significant chemokines with progression-free survival (PFS).** (A) CCL15; (B) MIF; (C) CCL24; (D) CXCL9; (E) CXCL13; (F) IFN-gamma; (G) IL-6; (H) IL-8; (I) IL-10.

**Figure S2. Kaplan-Meier survival analysis of 5 significant chemokines with overall survival (OS) and time-dependent operating characteristic curve (ROC) of CCL20.** (A) IL-8; (B) CCL24; (C) CXCL9; (D) CXCL13; (E) IL-6; Time-dependent operating ROC of CCL20 (F) for progression-free survival (PFS); (G) for OS.

**Figure S3. The functional enrichment and TCGA cohort analysis of chemokines related to crizotinib efficacy.** (A) Biological processes (BPs) enriched among the interaction molecules of 12 chemokines; (B) Angiogenesis-related BPs enriched among the interaction molecules by Cytoscape; (C) The mRNA expression of 4 chemokines between lung adenocarcinoma (LUAD), lung squamous carcinoma (LUSC), and normal tissue from the GEPIA database; (D) Kaplan-Meier curves of disease-free survival (DFS) and overall survival (OS) for CCL20 mRNA expression in TCGA cohort. * p＜0.05.

**Figure S4. The functional enrichment and GEPIA analysis of significant genes and the results of cell apoptosis assay.** (A) Angiogenesis-related BPs enriched among the differentially expressed genes from GSE94089 dataset by Cytoscape; (B) Effect of si-CCL2, si-CCL20, si-CCL24, and si-CX3CL1 treatments on cell apoptosis in H3122CR; (C) GEPIA correlation analysis for CCL20 with VEGFA, IL6, CCL2, and CCL24. ns: not significant.
